# Studying Interactions between 2’-O-Me-Modified Inhibitors and MicroRNAs Utilizing Microscale Thermophoresis

**DOI:** 10.1016/j.omtn.2019.08.019

**Published:** 2019-08-28

**Authors:** Markus Herkt, Sandor Batkai, Thomas Thum

**Affiliations:** 1 Institute of Molecular and Translational Therapeutic Strategies (IMTTS), Hannover Medical School (MHH), Hannover, Germany

**Keywords:** microscale thermophoresis, microRNA inhibitors, high-affinity binding

## Abstract

Besides the acquisition of pharmacokinetic parameters of antisense oligonucleotide microRNA (miRNA) inhibitors, such as measuring *in vivo* concentration, their pharmacodynamic characteristics are also of interest. An emerging and straightforward method for studying molecular interactions is microscale thermophoresis (MST). This technique makes it possible to study interactions between miRNAs and various oligonucleotide inhibitors, independent of the chemical modifications of the inhibitors or their respective target structure, with very little sample volume required compared to competitive techniques, such as surface plasmon resonance (SPR) and isothermal titration calorimetry (ITC). Interaction studies between these inhibitors and their respective target structures were performed, and they allowed the assessment of binding characteristics and parameters, such as EC_50_ for a number of these inhibitors, with little effort. Furthermore, MST could be utilized for obtaining kinetic binding data of the Argonaute-2 protein with a miRNA, which showed a possible RNA-induced silencing complex (RISC)-mediated turnover of inhibited miRNAs.

## Introduction

**Figure 1:**
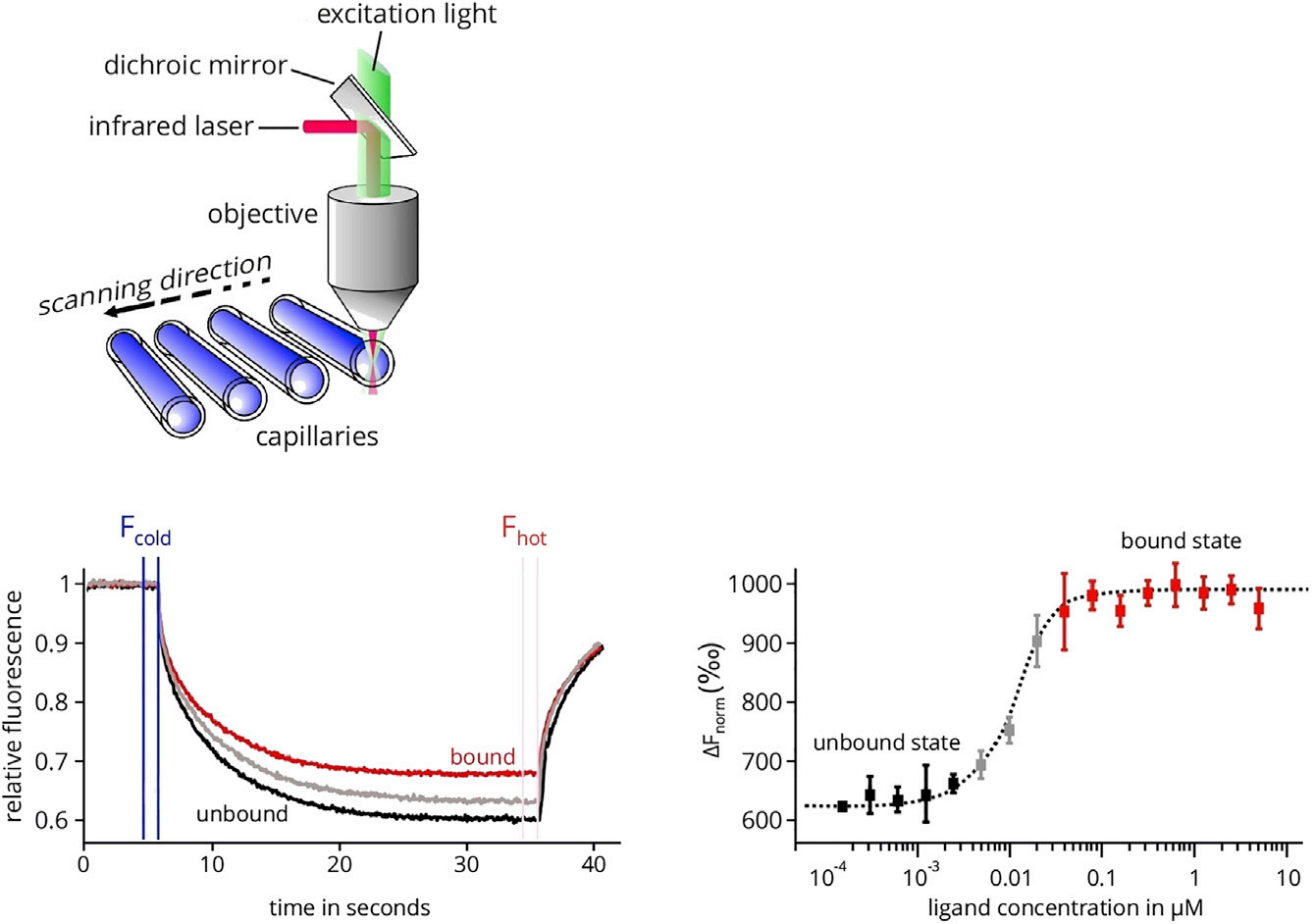
Physical Principle of MST Potential interaction partners are incubated in a physiological buffer and filled into a glass capillary. At a specific temperature, an infrared laser generates a temperature gradient. Interaction partners who have formed a complex move slower in this gradient than free interaction partners. A binding curve can be calculated from the difference between the fluorescence signals of both possible states, from which a binding constant can be derived. The figure has been modified and originally provided by NanoTemper Technologies, Munich.^9^

Noncoding RNAs have recently been identified as powerful targets to combat many different diseases.^1^ Indeed, microRNA (miRNA) therapeutics have been developed into the clinical stage where they have been successfully used in clinical trials.^2^ However, the development of effective and safe miRNA therapeutics is still a challenge, and many hurdles during their development and optimization have to be overcome. For instance, to assess the pharmacokinetic and pharmacodynamic properties of these drugs, qualitative as well as quantitative techniques are urgently needed. To date, a large variety of techniques are available, such as X-ray crystallography, nuclear magnetic resonance (NMR), mass spectrometry (MS), surface plasmon resonance (SPR), isothermal titration calorimetry (ITC), and thermal shift assays.^3^, ^4^ Those methods vary in terms of high-throughput ability, complexity, and the amount of sample needed. Ideally, those assays are very robust and reproducible, and they possess a low sample consumption. One emerging method to assess pharmacological parameters, such as the dissociation constant (K_D_), is microscale thermophoresis (MST). The basic mode of action of an MST experiment is illustrated below (Figure 1).

The flow of molecules induced by a temperature gradient in an aqueous solution is called the Ludwig-Soret effect. It describes the relationship between this heat flow and mass flow of molecules, which results in a local non-equilibrium, with complex size, hydration state, and charge state being the main driving forces for this flow of molecules.^5^ Thus, the movement of molecules in this temperature gradient can be described as a linear drift response.^6^ The Soret coefficient S_Ti_ is defined by S_Ti_ = D_Ti_/D_i_, with D_Ti_ being the thermophoretic mobility and D_i_ the diffusion coefficient.

$${\text{c}}_{\text{Ti}}{=\text{ c}}_{\text{i}}\;\text{exp}\;\left({\;-\text{ S}}_{\text{Ti}}\text{dT}\right)$$

Typically, the concentration c_Ti_ is lower than the initial concentration c_i_, which means a diffusion of molecules away from the source of the temperature gradient during a thermophoresis event. Thus, the thermophoretic mobility D_Ti_ and the Soret coefficient are typically positive.^7^

During a binding event, at least one of these parameters can vary, which renders MST to be a technique for analyzing several kinds of molecular interactions. In this study, we determined the binding event as well as the binding affinity of several chemically modified anti-miR oligonucleotides to their respective target mRNAs. MST turned out to be the ideal method to exactly quantify the EC_50_ of these interactions, and it proved to be a sensitive and robust approach for this kind of assay.

## Results

### The Influence of Chemical Modifications on Binding Affinity

**Figure 2:**
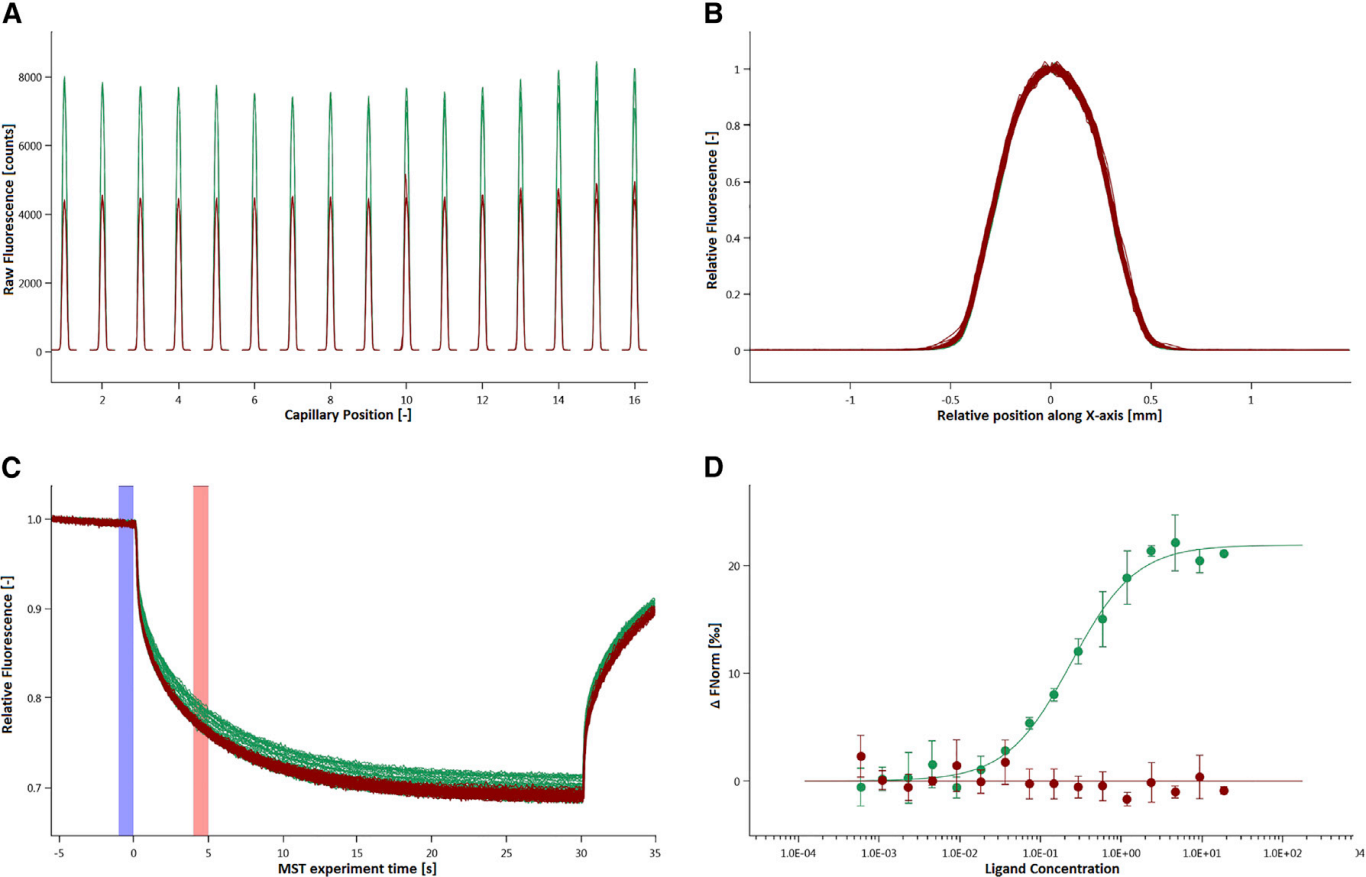
MST Analysis of OMe132_S5 Interacting with miRNA132 (A) Green peaks, OMe132_S5 versus miRNA132; red peaks, OMeScr versus miRNA132. (B) Green shapes, OMe132_S5 versus miRNA132; red shapes, OMeScr versus miRNA132. (C) Relative fluorescence (RF) between the bound and unbound state was determined over a time period of 35 s with 30 s MST-on time for evaluation. The blue bar indicates the ΔRF before temperature gradient was applied, whereas the red bar shows the ΔRF during the thermophoresis. Green traces, OMe132_S5 versus miRNA132; red traces, OMeScr versus miRNA132. (D) The EC_50_ was determined for this interaction employing standard data analysis with MO.Affinity Analysis Software. The interaction was plotted against OMeScr versus miRNA132 as a negative control. The graphs display data from 3 independent measurements. Error bars represent the SD. Green dots, OMe132_S5 versus miRNA132; red dots, OMeScr versus miRNA132.

Apart from sequence homology, other factors also have an influence on the binding affinity. Especially chemical modifications of single nucleotides can influence binding affinity, by changing the sterics or by creating additional interactions like hydrogen bonds between the nucleotides. For this reason, an anti-miRNA132 (antimiR132) library was designed, which besides sequence homology also contained different chemical modifications. 2’-O-Methyl modification (OMe) was chosen as ribose modification to study its influence on binding affinity (Table 1). The capillary scan, capillary shape, MST traces, and the binding curve of OMe132_S5 with miRNA132 are shown as examples (Figure 2), while the remaining binding curves can be found in the Supplemental Information.

**Table 1:** Oligonucleotides Used in This Study

Substance	Specifications
miRNA132_Cy5	(Cy5)-(NHC6)-mU-ps-mA-ps-mA-ps-mC-ps-mA-ps-mG-ps-mU-ps-mC-ps-mU-ps-mA-ps-mC-ps-mA-ps-mG-ps-mC-ps-mC-ps-mA-ps-mU-ps-mG-ps-mG-ps-mU-ps-mC-ps-mG; m_average_ = 7821.4 Da ± 0.05%; ε = 228400 L/mol*cm; purity > 85%; AXO LABS
miRNA24_Cy5	(Cy5)-(TEG)-mU-ps-mG-ps-mG-ps-mC-ps-mU-ps-mC-ps-mA-ps-mG-ps-mU-ps-mU-ps-mC-ps-mA-ps-mG-ps-mC-ps-mA-ps-mG-ps-mG-ps-mA-ps-mA-ps-mC-ps-mA-ps-mG; Eurogentec Deutschland
OMeScr	mA-ps-mC-ps-mG-ps-mU-ps-mC-ps-mU-ps-mA-ps-mU-ps-mA-ps-mC-ps-mG-ps-mC-ps-mC-ps-mC-ps-mA; m_average_ = 5130.2 Da ± 0.05%; ε = 145500 L/mol*cm; purity > 85%; AXO LABS
OMe132_S1	mA-ps-mU-ps-mG-ps-mG-ps-mC-ps-mU-ps-mG-ps-mU-ps-mA-ps-mG-ps-mA-ps-mC-ps-mU-ps-mG-ps-mU-ps-mU; purity > 85%; AXO LABS
OMe132_S2	mA-ps-mT-ps-mG-ps-mG-ps-mC-ps-mT-ps-mG-ps-mT-ps-mA-ps-mG-ps-mA-ps-mC-ps-mT-ps-mG-ps-mT-ps-mT; purity > 85%; AXO LABS
OMe132_S3	m(5-MeC)-ps-mT-ps-mG-ps-m(5-MeC)-ps-mT-ps-mG-ps-mA-ps-mA-ps-m(5-MeC)-ps-mT-ps-mG-ps-mA-ps-mG-ps-m(5-MeC)-ps-m(5-MeC); purity > 85%; AXO LABS
OMe132_S4	m(2-aminoA)-ps-mU-ps-mG-ps-mG-ps-mC-ps-mU-ps-mG-ps-mU-ps-m(2-aminoA)-ps-mG-ps-m(2-aminoA)-ps-mC-ps-mU-ps-mG-ps-mU-ps-mU; purity > 85%; AXO LABS
OMe132_S5	m(2-aminoA)-ps-mT-ps-mG-ps-mG-ps-m(5-MeC)-ps-mT-ps-mG-ps-mT-ps-m(2-aminoA)-ps-mG-ps-m(2-aminoA)-ps-m(5-MeC)-ps-mT-ps-mG-ps-mT-ps-mT; purity > 85%; AXO LABS
OMe24	mC-ps-mT-ps-mG-ps-mC-ps-mT-ps-mG-ps-mA-ps-mA-ps-mC-ps-mT-ps-mG-ps-mA-ps-mG-ps-mC-ps-mC; Eurogentec Deutschland
OMe24_1	mC-ps-mU-ps-mG-ps-mC-ps-mU-ps-mG-ps-mA-ps-mA-ps-mC-ps-mU-ps-mG-ps-mA-ps-mG-ps-mC-ps-mC; Eurogentec Deutschland
OMe24_2	m(5-MeC)-ps-mT-ps-mG-ps-m(5-MeC)-ps-mT-ps-mG-ps-mA-ps-mA-ps-m(5-MeC)-ps-mT-ps-mG-ps-mA-ps-mG-ps-m(5-MeC)-ps-m(5-MeC); Eurogentec Deutschland
OMe24_3	mC-ps-mT-ps-mG-ps-mC-ps-mT-ps-mG-ps-m(2-aminoA)-ps-m(2-aminoA)-ps-mC-ps-mT-ps-mG-ps- m(2-aminoA)-ps-mG-ps-mC-ps-mC; Eurogentec Deutschland
OMe24_4	m(5-MeC)-ps-mU-ps-mG-ps-m(5-MeC)-ps-mU-ps-mG-ps-mA-ps-mA-ps-m(5-MeC)-ps-mU-ps-mG-ps-mA-ps-mG-ps-m(5-MeC)-ps-m(5-MeC); Eurogentec Deutschland
OMe24_5	mC-ps-mU-ps-mG-ps-mC-ps-mU-ps-mG-ps-m(2-aminoA)-ps-m(2-aminoA)-ps-mC-ps-mU-ps-mG-ps-m(2-aminoA)-ps-mG-ps-mC-ps-mC; Eurogentec Deutschland
OMe24_6	m(5-MeC)-ps-mT-ps-mG-ps-m(5-MeC)-ps-mT-ps-mG-ps-m(2-aminoA)-ps-m(2-aminoA)-ps-m(5-MeC)-ps-mT-ps-mG-ps-m(2-aminoA)-ps-mG-ps-m(5-MeC)-ps-m(5-MeC); Eurogentec Deutschland
OMe24_7	m(5-MeC)-ps-mU-ps-mG-ps-m(5-MeC)-ps-mU-ps-mG-ps-m(2-aminoA)-ps-m(2-aminoA)-ps-m(5-MeC)-ps-mU-ps-mG-ps-m(2-aminoA)-ps-mG-ps-m(5-MeC)-ps-m(5-MeC); Eurogentec Deutschland
OMe24_8	m(5-MeC)-ps-mT-ps-mG-ps-mC-ps-mT-ps-mG-ps-mA-ps-mA-ps-mC-ps-mT-ps-mG-ps-mA-ps-mG-ps-mC-ps-m(5-MeC); Eurogentec Deutschland
OMe24_9	m(5-MeC)-ps-mU-ps-mG-ps-mC-ps-mU-ps-mG-ps-mA-ps-mA-ps-mC-ps-mU-ps-mG-ps-mA-ps-mG-ps-mC-ps-m(5-MeC); Eurogentec Deutschland
OMe24_10	mC-ps-mT-ps-m(2-aminoA)-ps-mC-ps-mT-ps-m(2-aminoA)-ps-mA-ps-mA-ps-mC-ps-mT-ps-m(2-aminoA)-ps-mA-ps-m(2-aminoA)-ps-mC-ps-mC; Eurogentec Deutschland
OMe24_11	m(2-aminoA)-ps-mT-ps-mG-ps-m(2-aminoA)-ps-mT-ps-mG-ps-mA-ps-mA-ps-m(2-aminoA)-ps-mT-ps-mG-ps-mA-ps-mG-ps-m(2-aminoA)-ps-m(2-aminoA); Eurogentec Deutschland

Nucleobases were modified as described: mC, 2′-O-methyl-cytosine; mT, 2′-O-methyl-thymine; mA, 2′-O-methyl-adenosine; mG, 2′-O-methyl-guanine; mU, 2′-O-methyl-uracile; ps, phosphorothioate; m(5-MeC), 2′-O-methyl-(5-methylcytosine); m(2-aminoA), 2′-O-methyl-(2-aminoadenine).

Pretests using standard- as well as premium-coated MST capillaries (NanoTemper Technology) were performed to test for adsorption of antimiR132 to capillary walls by analyzing capillary scans recorded by the Monolith NT.115_Pico_ prior to MST experiments. antimiR132 did not self-aggregate or adsorb to capillary walls in MST buffer, including 0.5% Tween 20 (NanoTemper Technologies), but it strongly self-aggregated when using premium-coated and standard-treated capillaries in the absence of 0.5% Tween 20 (data not shown). Thus, the reproducibility of MST signals was low. For subsequent experiments, standard-treated capillaries and MST buffer with 0.5% Tween 20 were used.

miRNA132 was labeled with Cy5 and used at a concentration of 3 nM, while OMe132_S5 was titrated in concentrations between 18.8 and 0.0006 nM. The variation between signal intensities was below 10% when adding 0.5% Tween 20 in MST buffer, which renders the use of a detergent being decisive for these measurements (Figure 2A), and the MST traces also show the typical course (Figure 2C). Also, a so-called Batman shape could not be observed, which would also have been an indication of major sticking to the capillary walls (Figure 2B). The binding curve for OMe132_S5 and miRNA132 also shows a very smooth transition from one plateau to the other, with an amplitude of Δ FNorm = 21.904 ‰ and the EC_50_ being 0.192 ± 0.0403 nM (Figure 2D). The EC_50_ values with their respective deviations of the other structural analogs are shown (Figure 4; Table 2).

**Table 2:** EC_50_ Values of OMe132/24 Structural Analogs Interacting with miRNA132/miRNA24

Oligonucleotide	[EC_50_] (nM)	±SD
OMe132_S1	0.574	0.0652
OMe132_S2	0.231	0.0601
OMe132_S3	0.395	0.0810
OMe132_S4	0.484	0.0552
OMe132_S5	0.216	0.0253
OMe24	2.94	0.328
OMe24_1	3.15	0.378
OMe24_2	1.04	0.377
OMe24_3	7.16	0.622
OMe24_4	8.32	0.848
OMe24_5	5.99	0.779
OMe24_6	3.22	0.235
OMe24_8	3.16	0.500
OMe24_9	5.31	2.84

### Applying MST to Self-Designed miRNA24 Inhibitors

**Figure 3:**
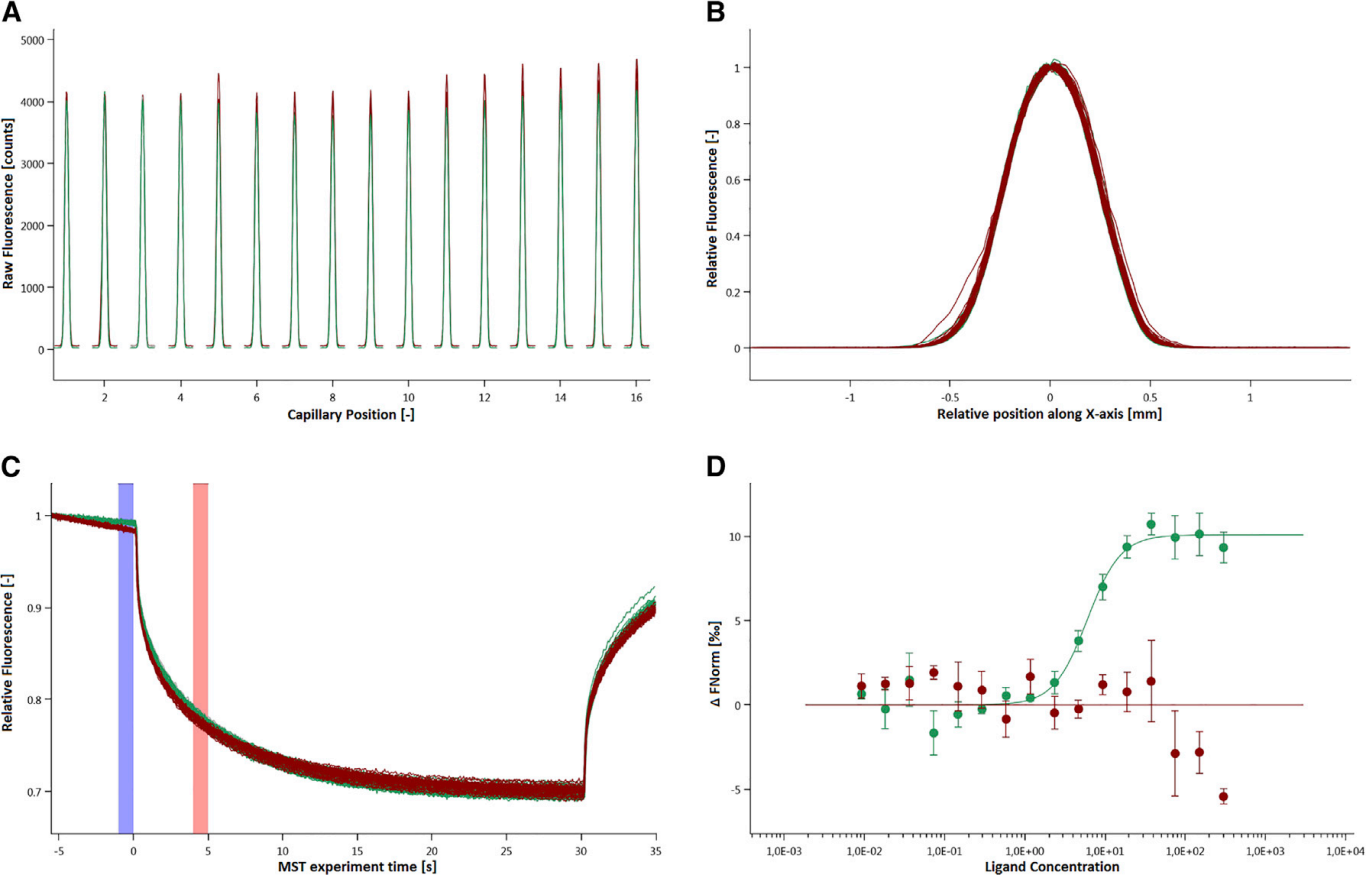
MST Analysis of OMe24_5 Interacting with miRNA24 (A) Green peaks, OMe24_S5 versus miRNA24; red peaks, OMeScr versus miRNA24. (B) Capillary shape shows no adsorption of analyte to the capillary surface. Green shapes, OMe24_S5 versus miRNA24; red shapes, OMeScr versus miRNA24. (C) Relative fluorescence (RF) between the bound and unbound state was determined over a time period of 35 s with 30 s MST-on time for evaluation. The blue bar indicates the ΔRF before temperature gradient of 2.5 K was applied, whereas the red bar shows the ΔRF during the thermophoresis. Green traces, OMe24_S5 versus miRNA24; red traces, OMeScr versus miRNA24. (D) The EC_50_ was determined for this interaction employing standard data analysis with MO.Affinity Analysis Software. The interaction was plotted against OMeScr versus miRNA24 as a negative control. The graphs display data from 3 independent measurements. Error bars represent the SD. Green dots, OMe24_S5 versus miRNA24; red dots, OMeScr versus miRNA24.

Another miRNA that plays a decisive role in angiogenesis and endothelial cell survival is miRNA24 (miRNA24), which was found to be enriched in cardiac endothelial cells and to be upregulated after ischemic injury.^8^ To extend the expertise in the design of oligonucleotide inhibitors to other targets, an antimiR24 (OMe24) library was designed in which the previously used chemical modifications were used to create the derivatives based on the miRNA24 primary sequence (Table 2). Figure 3 gives examples of a capillary scan, capillary shape, and MST traces of an interaction of OMe24_5 with miRNA24 as quality controls, while the binding curve shows an interaction between these two interaction partners (Figure 3). Binding curves of the other candidates can be found in the Supplemental Information.

Inhibitors based on OMe chemistry could also be designed for miRNA24, with which interaction studies could be performed using MST without changing the key parameters of the assay, which again proves the robustness of this method. miRNA24 was labeled with Cy5 and used at a concentration of 3 nM, while OMe24_S5 was titrated in concentrations between 300 and 0.0092 nM. All quality characteristics such as the fluorescence intensity, whose deviation in the capillary scan is less than 10%, or the MST traces, which show a normal course, are present (Figures 3A and 3C). Only the capillary shape shows small irregularities for single capillaries, which still leaves no doubt about the interaction between OMe24_5 and miRNA24, as the binding curve shows (Figure 3B). The amplitude is of Δ FNorm = 10.087 ‰ while the EC_50_ was calculated at 5.989 ± 0.779 nM (Figure 3D).

**Figure 4:**
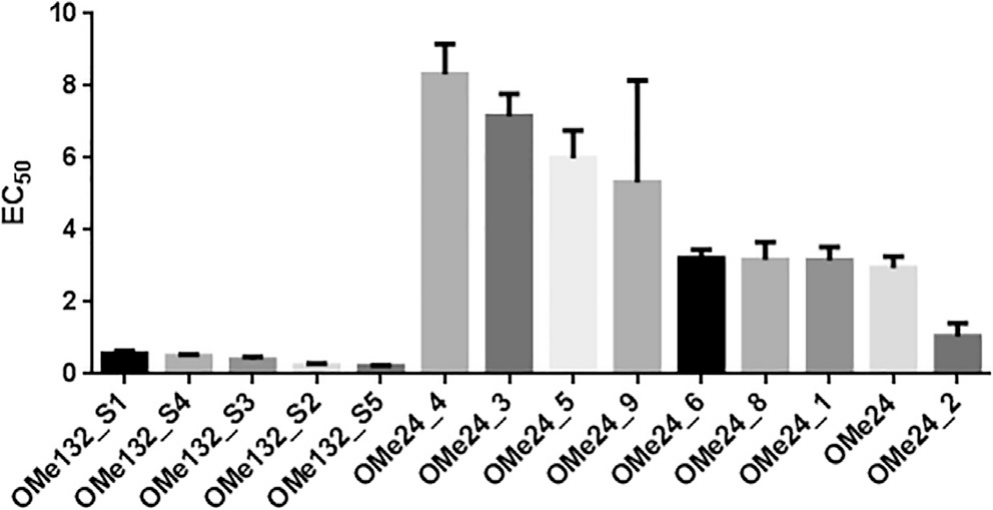
Comparision between OMe132 Analogs EC_50_ (nM) of OMe132 analogs were compared to illustrate the influence of chemical modification on binding affinity. The EC_50_ for the OMe132 analogs varies between 0.0625 and 0.0253 nM, whereas the EC_50_ for the OMe24 analogs varies between 8.32 and 1.04 nM. Both datasets were determined employing standard data analysis with MO.Affinity Analysis Software. The graphs display data from 3 independent measurements. Error bars represent the SD. OMe24_7; OMe24_10; OMe24_11, non-binder.

The EC_50_ values with their respective deviations of the other structural analogs are shown below (Figure 4; Table 1). Structural analogs were titrated in concentrations between 1,200 and 0.0366 nM (OMe132_S1 – OMe132_S4) and 18.8 and 0.0006 nM (OMe132_S5).

### The Role of AGO2 Catalytic Domain as Part of RISC in Possible miRNA Degradation

**Figure 5:**
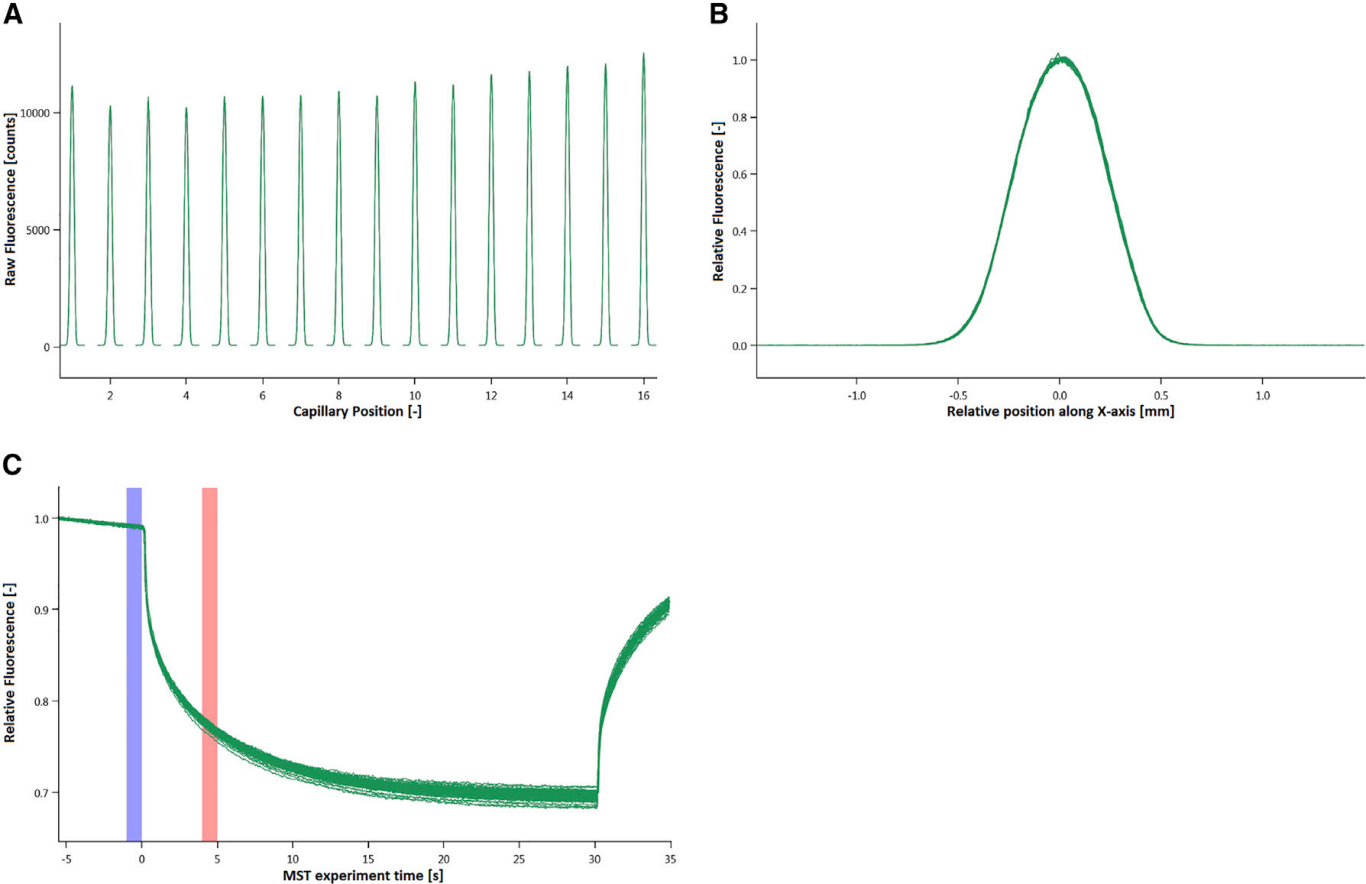
Quality Control of Argonaute 2 MST Data (A) The raw fluorescence signal was applied to the ordinate in arbitrary units, while the relative capillary position was applied to the abscissa. Green peaks, OMe132 + miRNA132 versus AGO2. (B) The relative fluorescence signal is applied to the ordinate in arbitrary units, while the capillary cross-section is applied to the abscissa. Green shape, OMe132 + miRNA132 versus AGO2. (C) Relative fluorescence (RF) between the bound and unbound state was determined over a time period of 35 s with 30 s MST-on time for evaluation. The blue bar indicates the ΔRF before temperature gradient of 2.5 K was applied, whereas the red bar shows the ΔRF during the thermophoresis. Green traces, OMe132_S5 + miRNA132 versus AGO2.

In addition to interactions between miRNAs and their inhibitors, interactions of miRNA versus inhibitor duplexes with proteins are also of interest. The Argonaute 2 protein (AGO2) is an important component of the RNA-induced silencing complex (RISC). The following study was performed to investigate whether the functionally active domain of the AGO2 protein could bind miRNA132+OMe132_S5 duplexes and catalytically convert these. Again the advantages of MST were used. To prove the quality of data, a capillary scan, capillary shape, and MST traces of an AGO2 and OMe132_S5 + miRNA132-duplex interaction are shown (Figure 5).

**Figure 6:**
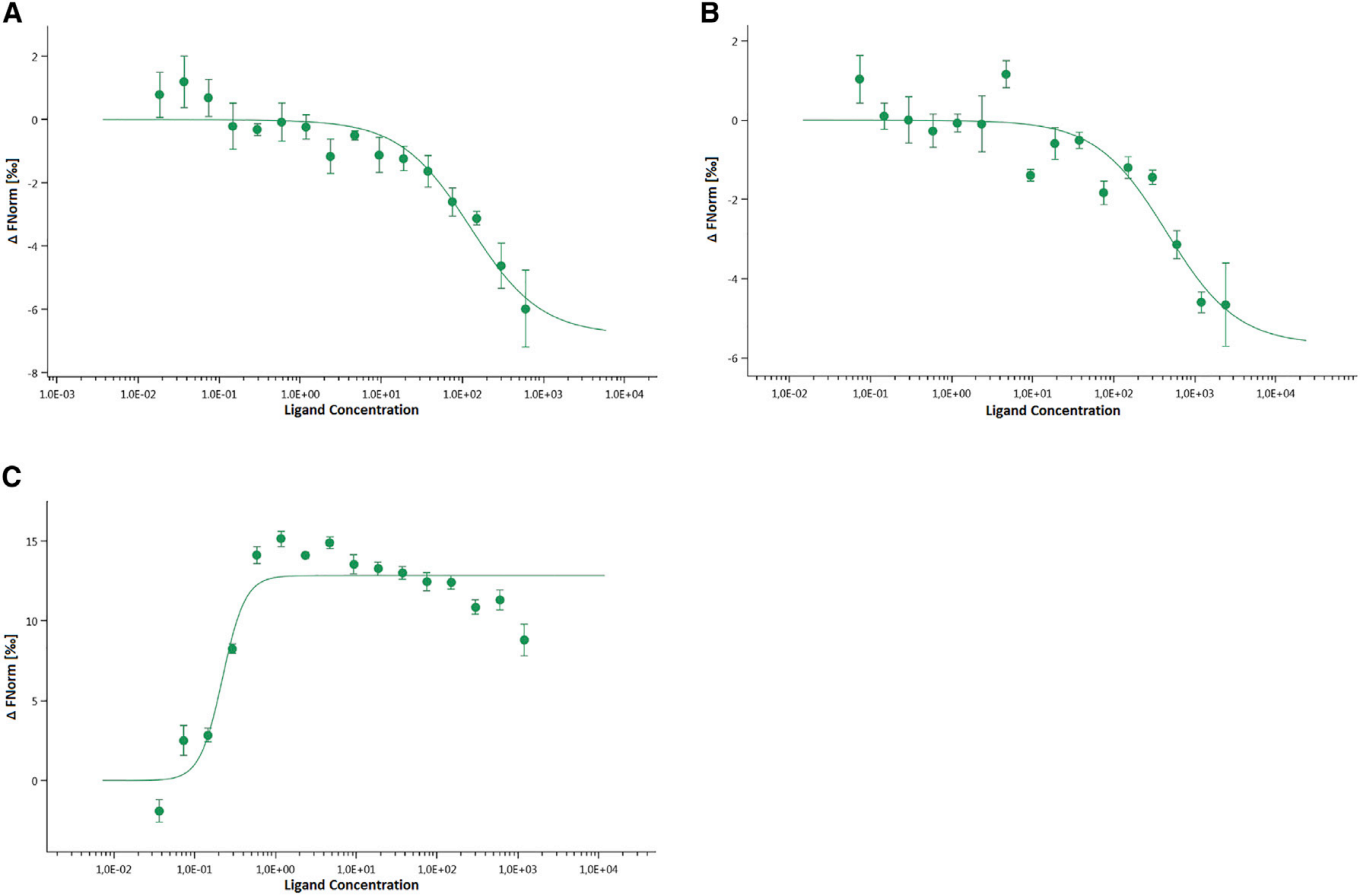
Monitoring Binding Events among AGO2, miRNA132, and OMe132 (A) A K_D_ of 124.88 nM was determined for this interaction employing standard data analysis with MO. Affinity Analysis Software. The graphs display data from 4 independent measurements. Error bars represent the SD. Green dots, OMe132_S5 + miRNA132 versus AGO2. (B) A K_D_ of 497.85 nM was determined for this interaction employing standard data analysis with MO.Affinity Analysis Software. The graphs display data from 5 independent measurements. Error bars represent the SD. Green dots, miRNA132 versus AGO2. (C) An EC_50_ of 0.2217 nM was determined for this interaction employing standard data analysis with MO.Affinity Analysis Software. The graphs display data from 5 independent measurements. Error bars represent the SD. Green dots, AGO2 + miRNA132 versus OMe132_S5.

Protein agglomerates were to be expected given the nature of large protein complexes or even catalytically active domains. Furthermore, an analyte sticking on the interior walls of the glass capillaries also was to be expected. Increasing the NaCl concentration to 300 mM prevented these issues and resulted in clean capillary scans and smooth MST traces (Figure 5). After proving that no agglomeration or analyte sticking occurred, binding curves of AGO2 with various binding partners were studied (Figure 6). Three possible interactions were tested. First OMe132_S5 was preincubated with miRNA132 and AGO2 titrated. In a second approach, AGO2 was titrated to miRNA132, while in a third setup, AGO2 was preincubated with OMe132_S5 and miRNA132 subsequently was added.

Binding curves with different characteristics could be determined for all three scenarios. For the interaction of the OMe132_S5/miRNA132 duplex with AGO2, the K_D_ was 124.88 nM, about a factor of 5 less than the interaction of AGO2 with miRNA132 alone. The latter amounted to 497.85 nM (Figures 6A and 6B). However, the interaction of the OMe132_S5+AGO2 complex with later addition of miRNA132 showed an EC_50_ of 0.2217 nM (Figure 6C).

**Figure 7:**
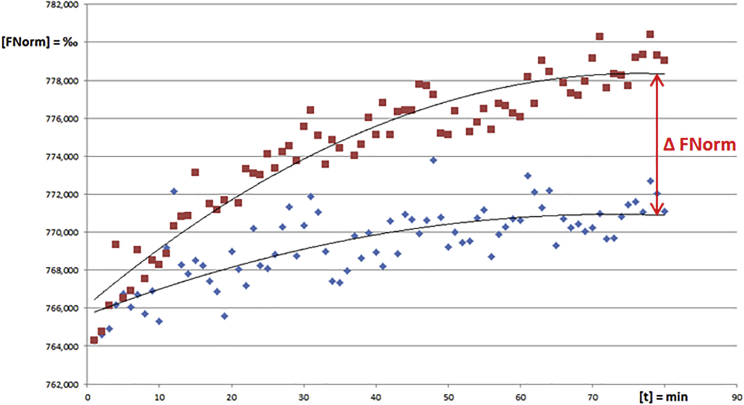
Possible Degradation of miRNA132-Inhibitor Complexes via Argonaute 2 Normalized fluorescence intensities [fNorm] = ‰ have been plotted versus the time [t] = min. Orange dots, OMe132_S5 + miRNA132 versus AGO2; blue dots, miRNA132 versus AGO2 (control).

Besides a binding event between AGO2 and a duplex of miRNA132 and its inhibitor, a possible metabolism and degradation event of antimiR132 was also of interest. This could give an indication of the fate of an administered oligonucleotide inhibitor, especially in view of the fact that modified oligonucleotides are resistant to exo- as well as endonuclease activity and, thus, possess a poor metabolization behavior. To answer this question using MST technology, 12 nM miRNA132 was preincubated with 12 nM OMe132_S5 to form a duplex (c_final_ = 6 nM), which should then serve as substrate for AGO2. As a negative control, AGO2 was titrated to 12 nM miRNA132 (c_final_ = 6 nM). Over a period of 80 min, the raw fluorescences were recorded, and the two interaction approaches against each other were plotted (Figure 7).

Looking at the two fluorescence sequences, it was noticeable that the increase in the raw fluorescence of the AGO2/+duplex interaction was significantly greater compared to the AGO2+miRNA132 interaction (negative control) over time. However, both entered a saturation phase after approximately 70 min, so that there was no further increase in fluorescence (Figure 7).

## Discussion

The study of biomolecular interactions is a basic prerequisite for the development of novel drugs, as many pharmacological target structures interact with other effector molecules and may trigger a biological effect that can cause a certain disease pattern. Several methods can be used to investigate biomolecular interactions. Depending on the application and the specific subject to investigate, different methods have proven their worth. SPR for instance is a label-free method that can be used to investigate not only specificity and affinity but also pharmacokinetic parameters, in particular protein-DNA and receptor-ligand interactions, which makes this method particularly suitable for drug discovery.^9^, ^10^, ^11^ An intrinsic disadvantage of SPR is the unspecific binding of non-interaction partners to the metal surface, which results in interference of the resulting signals. In addition, current SPR instruments are still very cumbersome and expensive, so there is still no greater dissemination of these instruments in medicine and science.^12^

As for ITC, it is performed at a constant temperature where one binding partner is present in a sample cell while the other binding partner is titrated. The energy released is compared with a reference cell, while the electrical power required to maintain a constant temperature difference between the two cells is a measure of the heat change to be determined. The total heat accumulated is normalized to the total ligand concentration and plotted against the total ligand concentration.^13^

A severe drawback for this technique is that a heat capacity change is due to many different effects, so that only in scenarios where free molecules change into a bound state without changing their hydration state or are being protonated give the exact quantity of ΔH, which is why ΔH is often referred to as apparent heat change ΔH_app_.

Thus, the method of choice in this study was MST. For all experiments, the assay was performed under the same conditions and parameters. The capillary scan, capillary shape, and the MST traces showed no deviations from the usual behavior, and a clear binding curve between antimiR132 and miRNA132 could be shown compared to antimiRScr (Figure 2). To test the robustness of the MST system, a small miRNA132 inhibitor library containing 5 structural analogs was examined for binding to miRNA132. All inhibitors showed a sub-nanomolar EC_50_, which justified the use of the NT.115 Pico in particular (Figure 4; Table 1). The sequence of inhibitors from less affinity to high affinity was very well matched with the type of chemical modifications of the individual library candidates. Uracil lacks a methyl group compared to thymine, so there was no additional stacking effect between the bases (OMe132_S1 versus OMe132_S2). This was achieved by using 5-methylcytosine, which resulted in a higher binding affinity (OMe132_S3). By derivatizing adenine to 2-aminoadenine, a third additional hydrogen bridge could be formed between adenine and thymine, resulting in an even higher binding affinity (OMe132_S4). By combining both modifications, i.e., 5-methylcytosine and 2-aminoadenine, the highest binding affinity could be achieved (OMe132_S5), which was shown as an example (Figure 2D). Particularly positive is the fact that MST was able to visualize these fine differences in the EC_50_, which was also due to the sensitivity of the NT.115 Pico.

**Figure 8:**
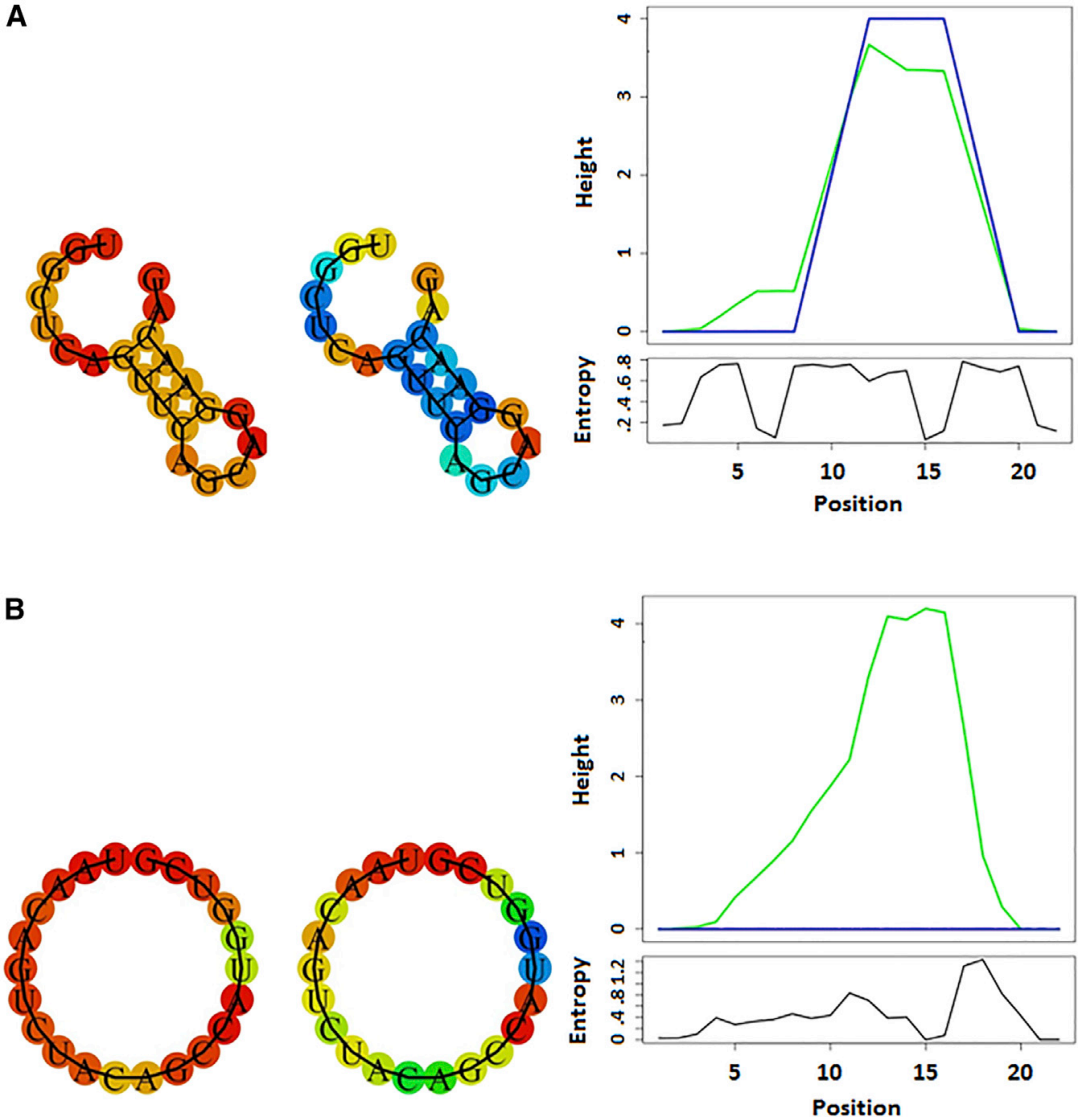
Predicted Secondary Structure of miRNA24 and miRNA132 (A) Secondary structures were predicted with the Vienna Web Services Tool based on the primary sequence of miRNA24. Left structure: basepair propabilities (blue, low; red, high); right structure: positional entropy (red, low; blue, high). Both color codes follow the visible light spectrum (blue → green → yellow → red). The mountain plot represents the secondary structure in a plot of height versus position, where the height m(k) is given by the number of base pairs enclosing the base at position k. Loops correspond to plateaus, helices to slopes. Plotting was performed utilizing the RNAfold Web Server. (B) Structures were predicted with the Vienna Web Services Tool based on the primary sequence of miRNA132. Left structure: basepair propabilities (blue, low; red, high); right structure: positional entropy (red, low; blue, high). Both color codes follow the visible light spectrum (blue → green → yellow → red). The mountain plot represents the secondary structure in a plot of height versus position, where the height m(k) is given by the number of base pairs enclosing the base at position k. Loops correspond to plateaus, helices to slopes. Plotting has been performed utilizing the RNAfold Web Server.

Based on previous findings regarding the influence of chemical modifications on the binding affinity of OMe132 inhibitors against miRNA132, an inhibitor library was designed to target miRNA24 containing permutations of the same chemical modifications. Again, MST could be utilized to determine EC_50_ values independently of the putative interaction partners and chemical modifications without changing any parameters on the assay, which was further proof of the robustness of this particular method (Figure 3). Binding curves and EC_50_ values could be determined for almost all candidates. As far as OMe24_10 and OMe24_11 were concerned, there was no sequence homology between these candidates and miRNA24. However, an attempt was made to compensate the missing sequence homology by chemical modifications in order to induce a binding to miRNA24. The artificial base 2-aminoadenine was intended to replace the missing hydrogen bridge that guanine builds up to cytosine in the target sequence and vice versa. It most likely didn’t work because the steric properties of the respective base in space were not taken into account. OMe24_7 and OMe24_9 on the other hand were a different story. Complete sequence homology prevailed between this candidate and miRNA24, and artificial bases such as 5-methylcytosine and 2-aminoadenine were used to increase the binding affinity. Nevertheless, no binding event took place between OM24_7 or OMe24_9, respectively, and miRNA24. If the primary sequence could not be the cause for the missing binding event between OMe24_7 or OMe24_9 and miRNA24, respectively, then the secondary structure should provide information about it (Figure 8A).

For miRNA24 a secondary structure could be predicted, so that a steric inhibition due to the predicted hairpin seemed plausible when binding to an inhibitor. However, the question arose why only some of the inhibitors that otherwise had sequence homology to miRNA24 had no affinity to their target structure. This question could only be answered by an intensive modeling of the different inhibitors in complex with miRNA24, which would have gone beyond the scope of this study. Nevertheless, further structural investigations of inhibitor design should be carried out to improve the pharmacokinetic properties of these inhibitors.

Furthermore, the binding affinity also did not seem to correlate with the type and influence of the specific modifications, as was the case with the OMe132 library (Table 3). Thus, the Vienna Web Services Tool was utilized to predict a possible secondary structure of miRNA132 (Figure 8B).

**Table 3:** Chemicals and Reagents Used in This Study

Substance	Specifications
CaCl_2_	dehydrated; Fluka
EDTA	Sigma-Aldrich
KCl	dehydrated; Fluka
MgCl_2_	dehydrated; Fluka
NaCl	chem^solute^
Tris	MP Biomedicals
Tris/HCl buffer	1.5 M; pH 6.8/8.8
Water	distilled; Millipore

Using this approach no secondary structure could be predicted for miRNA132, so that the primary sequence and the type of chemical modifications alone were most likely the cause for the different binding affinities of the OMe132 library candidates in comparison to the OMe24 candidates.

Besides investigations on the binding of different miRNA132 inhibitors to their target structure and the dependence of the binding affinity on the chemical and structural proportions of these inhibitors, the question whether these inhibitors are metabolized and degraded after they have recognized and bound their target structure was also of interest. The RISC plays a decisive role in miRNA degradation after it bound its target mRNA. During this process the miRNA serves as a guide, which is then recognized by the RISC. Thus, it seemed reasonable to check to what extent the catalytically active domain of RISC, namely AGO2, is able to recognize and degrade miRNA+inhibitor complexes. Since in this study it was the first time to look at RNA-protein interaction, some modifications had to be made to the assay. In particular, the NaCl concentration had to be drastically increased to ensure the structural integrity of the AGO2 subdomain and to obtain clear capillary scan, capillary shapes, and MST traces (Figure 5).

After the stability of the assay was ensured, measurements were taken to observe the interaction among AGO2, miRNA132, and OMe132_S5 (inhibitor) from all perspectives. Interactions between AGO2 versus miRNA132+OMe132_S5, AGO2 versus miRNA132, and AGO2+miRNA132 versus OMe132_S5 could be proven (Figure 6). As for the AGO2+miRNA132 versus OMe132_S5 setup, it was likely that most of the interaction took place between miRNA132 and its inhibitor. This suspicion was confirmed by the fact that the measured EC_50_ was comparable to the previously measured miRNA132 versus OMe132_S5 interaction (Figures 6C and 2). This was to be expected since AGO2 should not have a binding site for artificial DNA-based miRNA inhibitors such as OMe132_S5. On the other hand, AGO2 has a binding site for miRNAs, which could be shown for the AGO2 versus miRNA132+OMe132_S5 interaction, with the K_D_ being in the three-digit nanomolar range but significantly higher compared to the miRNA132 versus OMe132_S5 interaction (Figures 6A and 6B). This indicated that the miRNA132+OMe132_S5 duplex was already formed and subsequently bound to AGO2. Interestingly, the K_D_ determined for AGO2 with miRNA132 alone was approximately 4 times higher compared to the AGO2 interaction with the duplex of miRNA132 and inhibitor.

Based on these results it had to be assumed that binding of an inhibitor to miRNA132 increased the affinity to AGO2. This may have been due to conformational changes of the AGO2 catalytic domain or an altered hydrodynamic radius, charge, and hydration state of the miRNA132+OMe132_S5 duplex. After a binding of the miRNA132+OMe132_S5 duplex to AGO2 could be shown, it was investigated whether and to what extent AGO2 was able to degrade this duplex utilizing MST (Figure 7). Since a miRNA works as a guide for a mRNA to be directed to the RISC, no turnover should be observed in the AGO2+miRNA132 setup. Interestingly, a slight increase in the signals of the AGO2+miRNA132 setup could be observed, whereby the slope of the curve differed considerably compared to the AGO2 versus miRNA132+OMe132_S5 setup like described previously in a similar experiment.^14^ The difference between the fluorescence probably likely derived from the degradation of the duplex, so that the fluorophore (Cy5) was released and quenching of the latter was reduced. This indicated that some degradation process might have been taking place. With these experimental setups, MST could be shown to display even miRNA versus protein interactions and, furthermore, catalytic activity of an important subdomain of the RISC by assessing raw data.

## Materials and Methods

### Experimental Setup

miRNA132 was synthesized *de novo* and labeled using a Cy5 fluorophore via an Amino-C6-linker (NHC6) by Axolabs and supplied lyophilized. After labeling, miRNA132 was eluted into physiological buffer, including 20 mM Tris (pH 7.4), 150 mM NaCl, 5 mM KCl, 1 mM CaCl_2_, 1 mM MgCl_2_, and 0.5% Tween 20, which was also used as assay buffer for MST experiments. miRNA132 was used as target at a concentration of 3 nM, while non-fluorescent anti-miRNA oligonucleotides (OMe132) analogs were titrated in a 1:1 dilution series (concentrations between 1,200 and 0.037 nM). The same procedure was repeated using miRNA24 and OMe24. Samples were loaded into Monolith NT.115_Pico_ MST standard-treated capillaries (NanoTemper Technologies) and measured using a Monolith NT.115_Pico_ and MO.Control software at 37°C (light-emitting diode [LED] or excitation power setting 10%, MST power setting 40%). Data were analyzed using MO.Affinity Analysis software (version 2.2.4, NanoTemper Technologies) at the standard MST-on time of 30 s.

## Author Contributions

M.H., performance of all experiments and writing of the first draft of the manuscript; S.B., idea and intellectual support of M.H. and the technology; T.T., organized funding of the project, and supervised M.H. and supported M.S. with writing.

## Conflicts of Interest

T.T.and S.B. are co-founders and shareholders of Cardior Pharmaceuticals. T.T. has filed and licensed patents about noncoding RNAs.

## Supplemental Information

Document S1. Figures S1–S16

Document S2. Article plus Supplemental Information
