# Supplementary material for: Studying Interactions between 2’-O-Me-Modified Inhibitors and MicroRNAs Utilizing Microscale Thermophoresis
Source: Mol Ther Nucleic Acids. 2019 Aug 28;18:259–68. doi: 10.1016/j.omtn.2019.08.019 (PMC6796726; doi:10.1016/j.omtn.2019.08.019)
Supplement: Document S1. Figures S1–S16 [file mmc1.pdf]

OMTN, Volume *18*

Supplemental Information

Studying Interactions between 2'-O-Me-Modified Inhibitors and MicroRNAs Utilizing Microscale Thermophoresis

Markus Herkt, Sandor Batkai, and Thomas Thum

Supplementary information

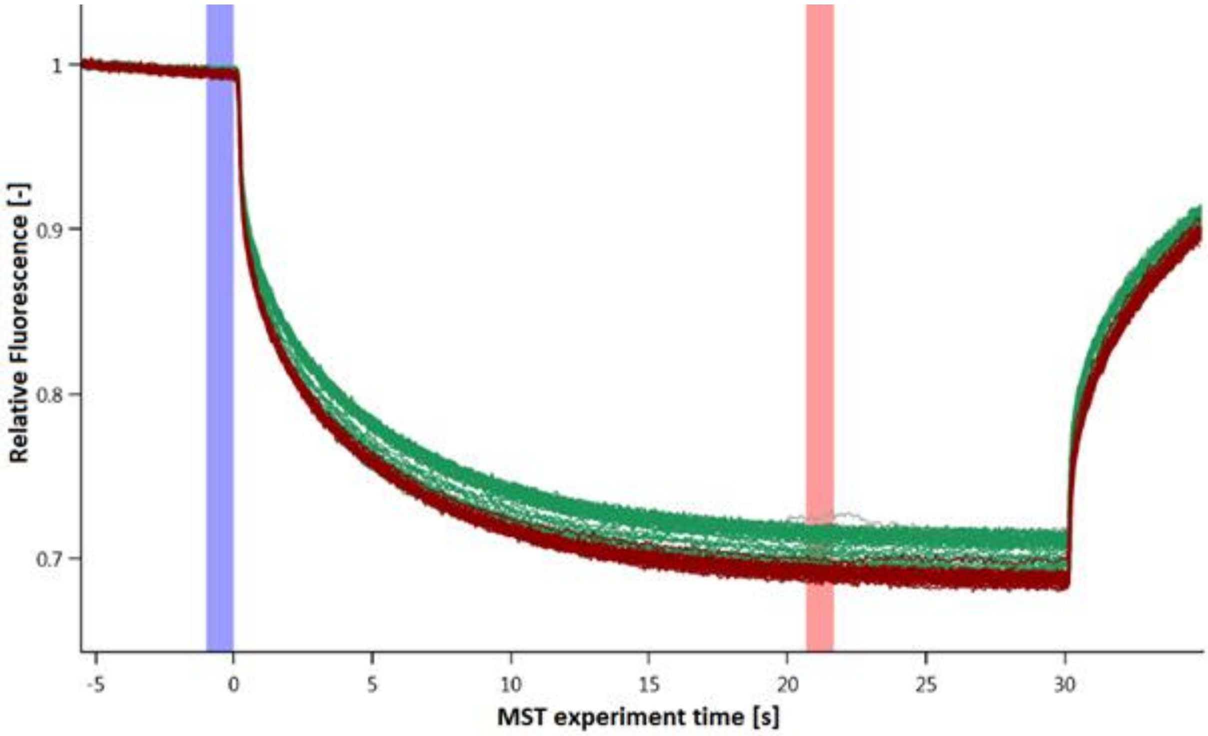

Supplementary Figure 1A| MST traces of OMe132_S1 interacting with miRNA132. Relative Fluorescence (RF) between the bound and unbound state was determined over a time period of 35 s with 30 s MST-on time for evaluation. The blue bar indicated the ΔRF before Temperature gradient of 2.5 K was applied, whereas the red bar shows the ΔRF during the thermophoresis. Green traces: OMe132_S1 vs. miRNA132; red traces: OMeScr vs. miRNA132.

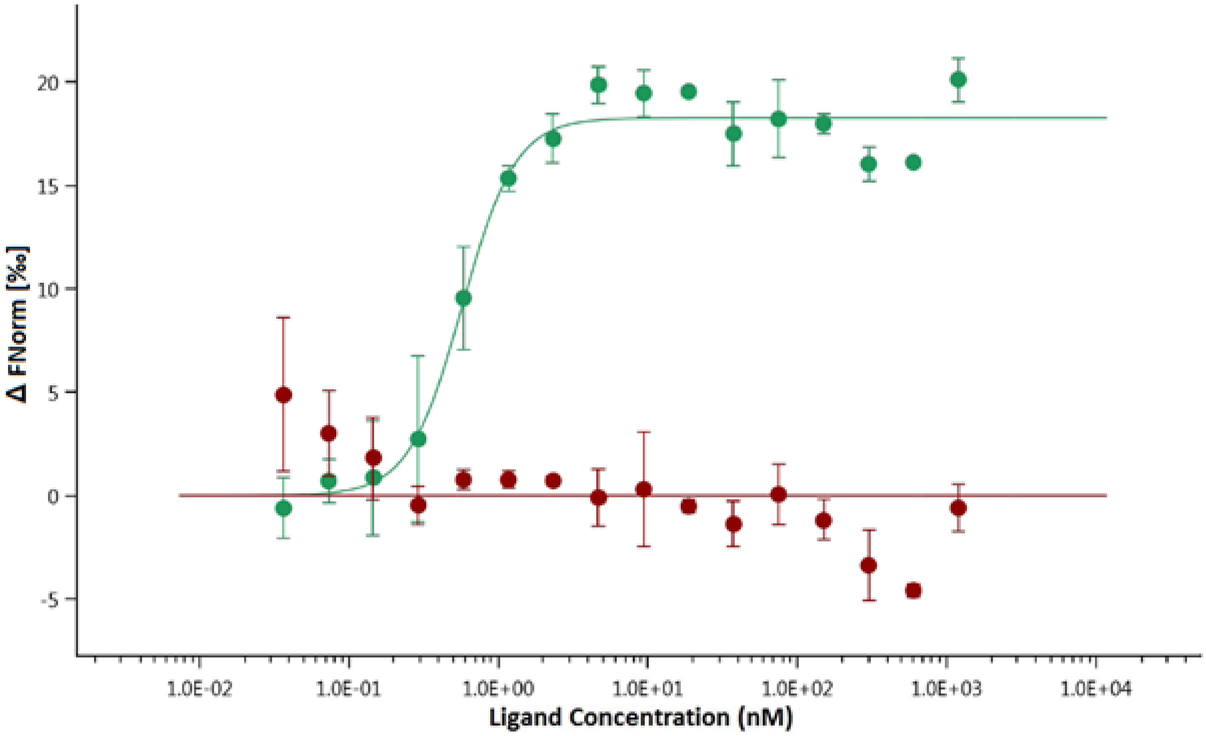

Supplementary Figure 2B| Interaction between OMe132_S1 and miRNA132. MiRNA132 was labeled with Cy5 and used at a concentration of 3 nM while OMe132_S1 was titrated in concentrations between 1200 and 0.0366 nM. A EC_{50} of 0.574 nM +/- 0.0652 nM was determined for this interaction employing standard data analysis with MO.Affinity Analysis Software. The interaction was plotted against OMeScr vs. miRNA132 as negative control. The graphs display data from 3 independent measurements. Error bars represent the standard deviation. Green dots: OMe132_S1 vs. miRNA132; red dots: OMeScr vs. miRNA132.

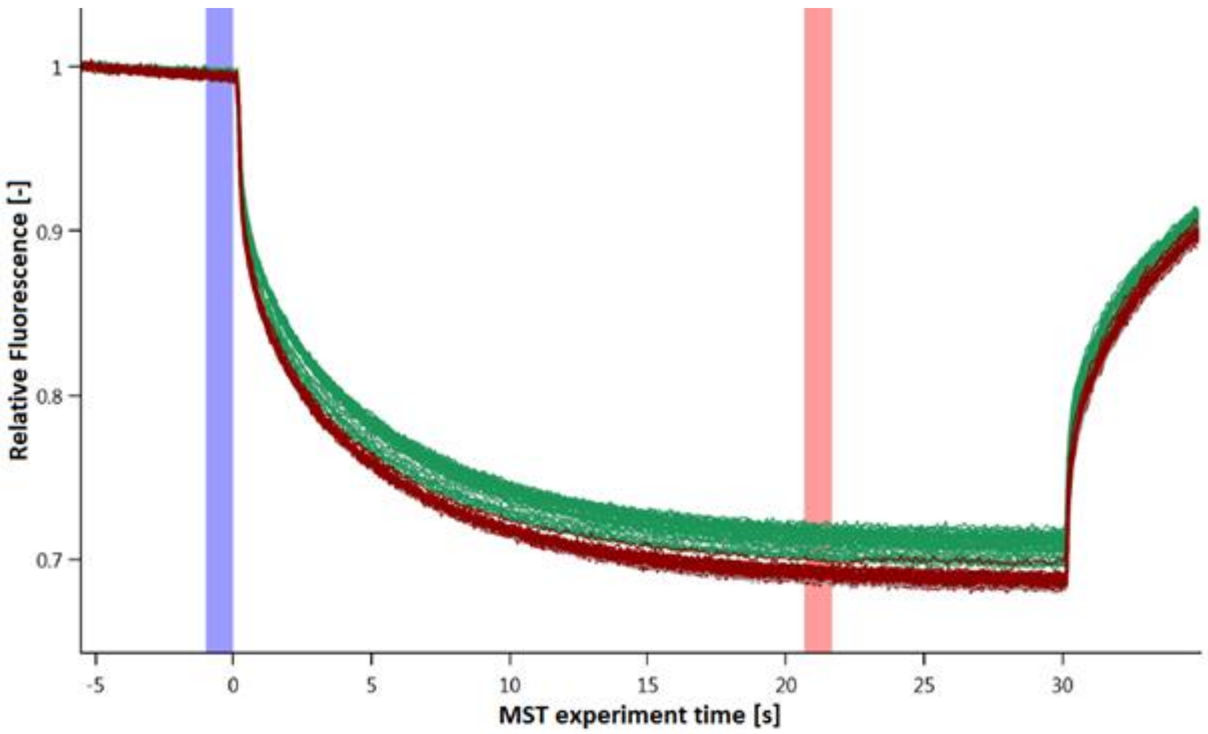

Supplementary Figure 3A| MST traces of OMe132_S2 interacting with miRNA132. Relative Fluorescence (RF) between the bound and unbound state was determined over a time period of 35 s with 30 s MST-on time for evaluation. The blue bar indicates the ΔRF before Temperature gradient of 2.5 K was applied, whereas the red bar shows the ΔRF during the thermophoresis. Green traces: OMe132_S2 vs. miRNA132; red traces: OMeScr vs. miRNA132.

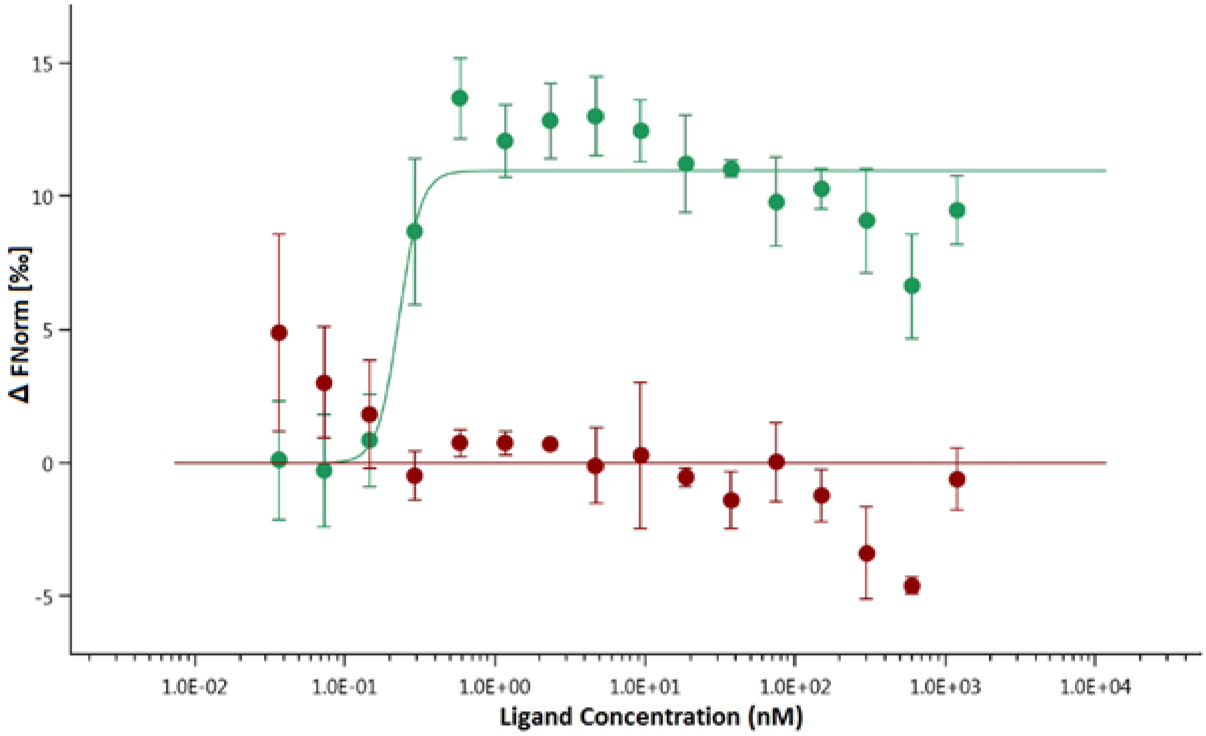

Supplementary Figure 3B| Interaction between OMe132_S2 and miRNA132. MiRNA132 was labeled with Cy5 and used at a concentration of 3 nM while OMe132_S1 was titrated in concentrations between 1200 and 0.0366 nM. A EC_{50} of 0.231 nM +/- 0.0601 nM was determined for this interaction employing standard data analysis with MO.Affinity Analysis Software. The interaction was plotted against OMeScr vs. miRNA132 as negative control. The graphs display data from 3 independent measurements. Error bars represent the standard deviation. Green dots: OMe132_S2 vs. miRNA132; red dots: OMeScr vs. miRNA132.

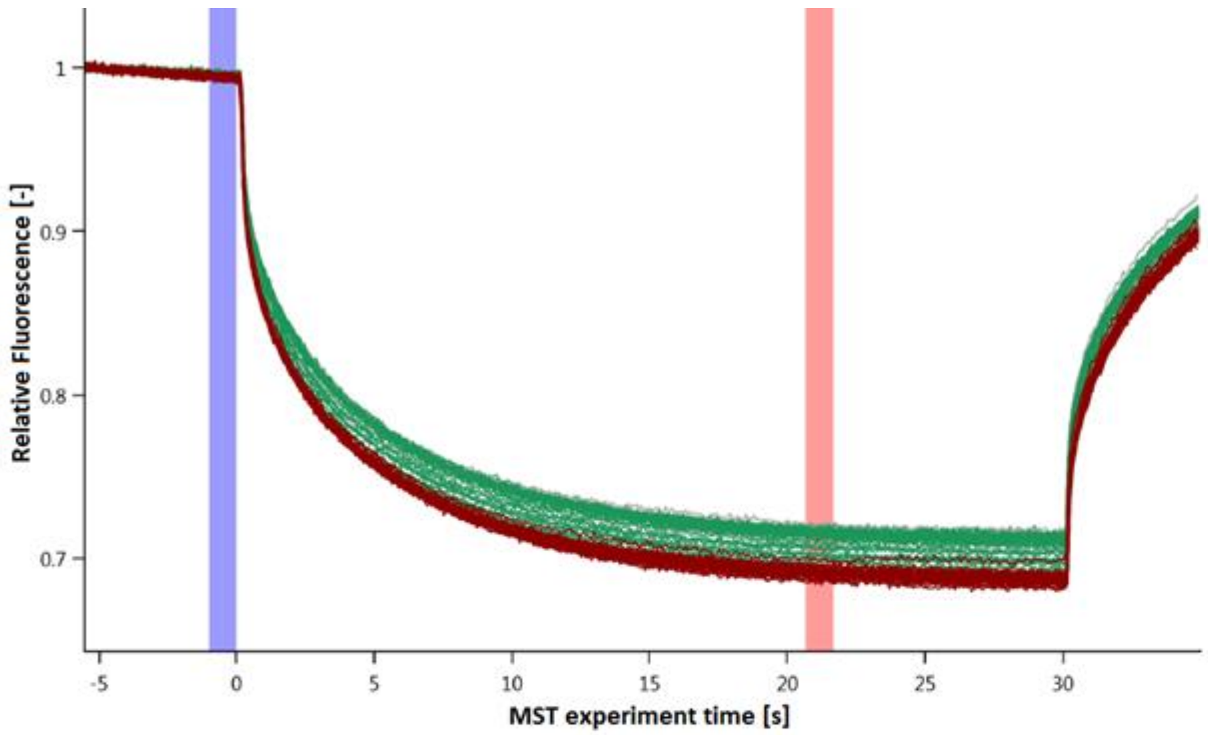

Supplementary Figure 4A| MST traces of OMe132_S3 interacting with miRNA132. Relative Fluorescence (RF) between the bound and unbound state was determined over a time period of 35 s with 30 s MST-on time for evaluation. The blue bar indicates the ΔRF before Temperature gradient of 2.5 K was applied, whereas the red bar shows the ΔRF during the thermophoresis. Green traces: OMe132_S3 vs. miRNA132; red traces: OMeScr vs. miRNA132.

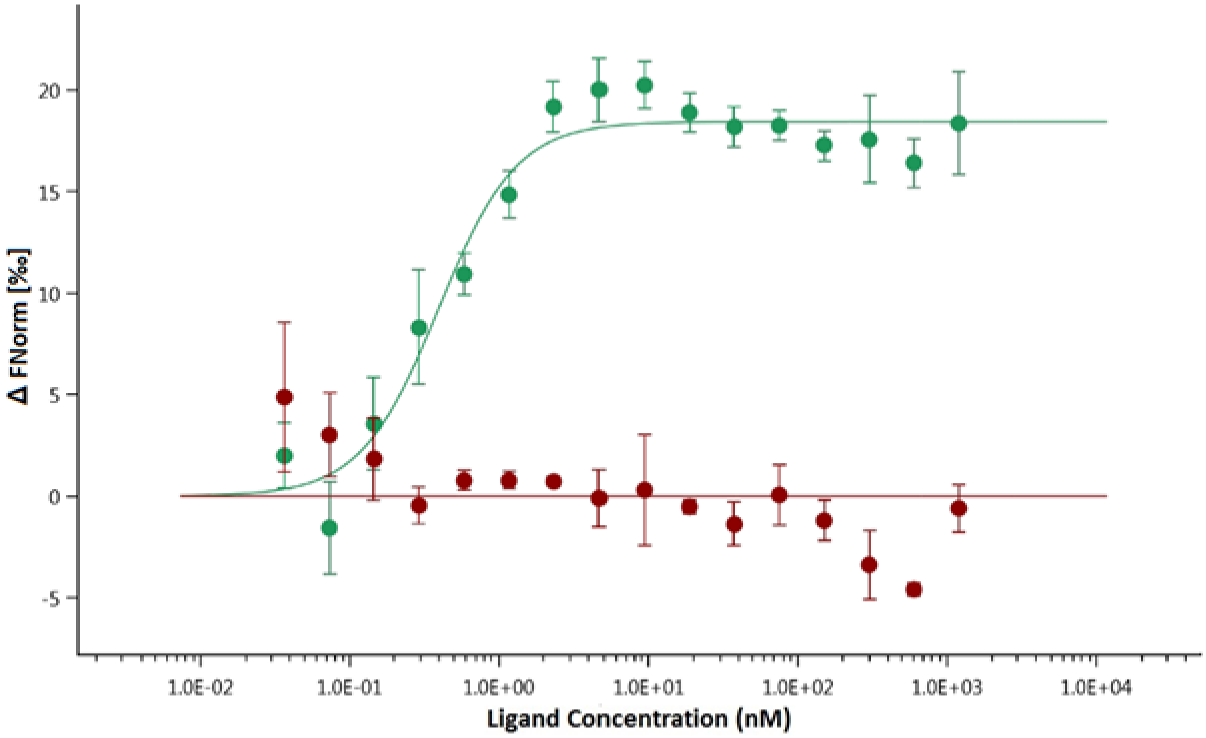

Supplementary Figure 4B| Interaction between OMe132_S3 and miRNA132. MiRNA132 was labeled with Cy5 and used at a concentration of 3 nM while OMe132_S1 was titrated in concentrations between 1200 and 0.0366 nM. A EC_{50} of 0.395 nM +/- 0.0810 nM was determined for this interaction employing standard data analysis with MO.Affinity Analysis Software. The interaction was plotted against OMeScr vs. miRNA132 as negative control. The graphs display data from 3 independent measurements. Error bars represent the standard deviation. Green dots: OMe132_S3 vs. miRNA132; red dots: OMeScr vs. miRNA132.

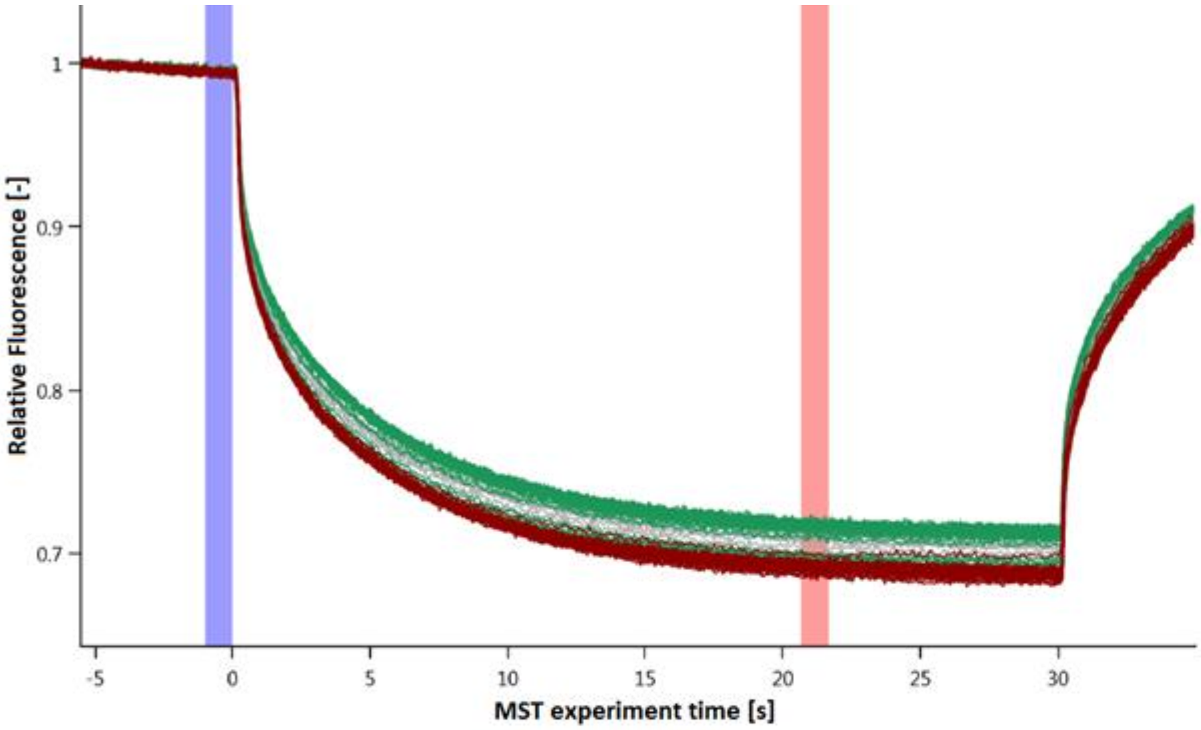

Supplementary Figure 5A| MST traces of OMe132_S4 interacting with miRNA132. Relative Fluorescence (RF) between the bound and unbound state was determined over a time period of 35 s with 30 s MST-on time for evaluation. The blue bar indicates the ΔRF before Temperature gradient of 2.5 K was applied, whereas the red bar shows the ΔRF during the thermophoresis. Green traces: OMe132_S4 vs. miRNA132; red traces: OMeScr vs. miRNA132.

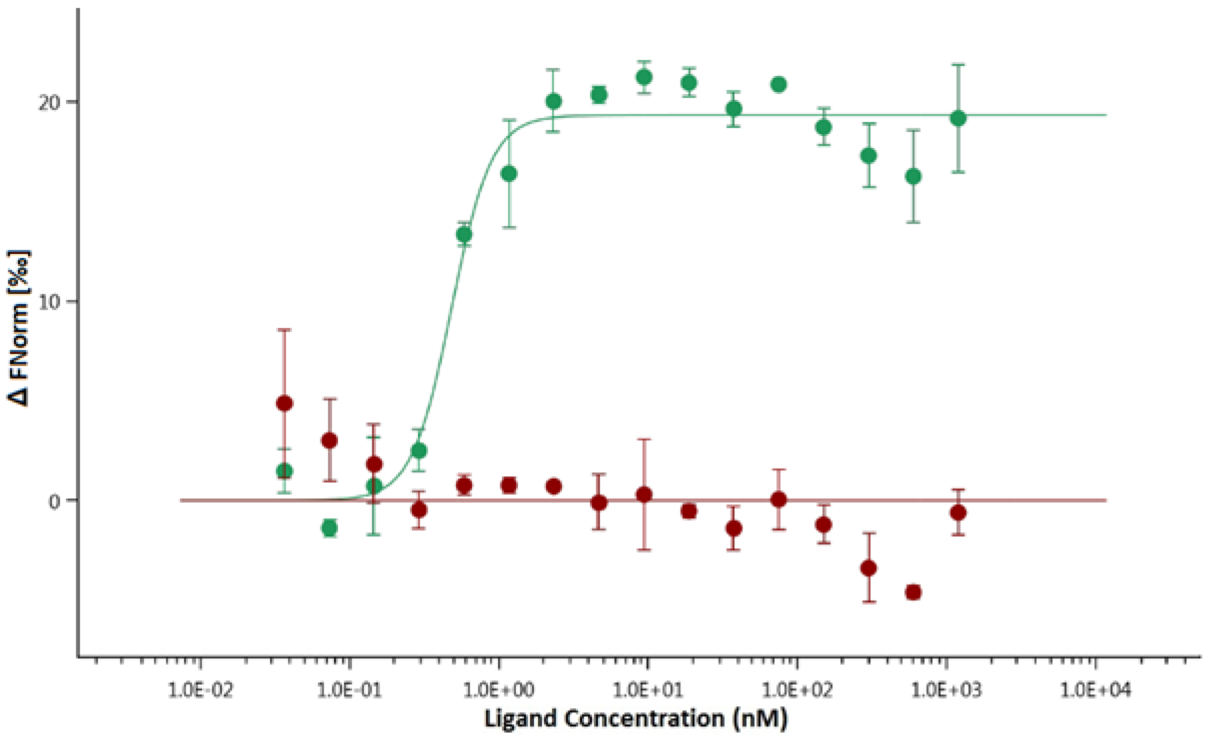

Supplementary Figure 5B| Interaction between OMe132_S4 and miRNA132. MiRNA132 was labeled with Cy5 and used at a concentration of 3 nM while OMe132_S1 was titrated in concentrations between 1200 and 0.0366 nM. A EC_{50} of 0.484 nM +/- 0.0553 nM was determined for this interaction employing standard data analysis with MO.Affinity Analysis Software. The interaction was plotted against OMeScr vs. miRNA132 as negative control. The graphs display data from 3 independent measurements. Error bars represent the standard deviation. Green dots: OMe132_S4 vs. miRNA132; red dots: OMeScr vs. miRNA132.

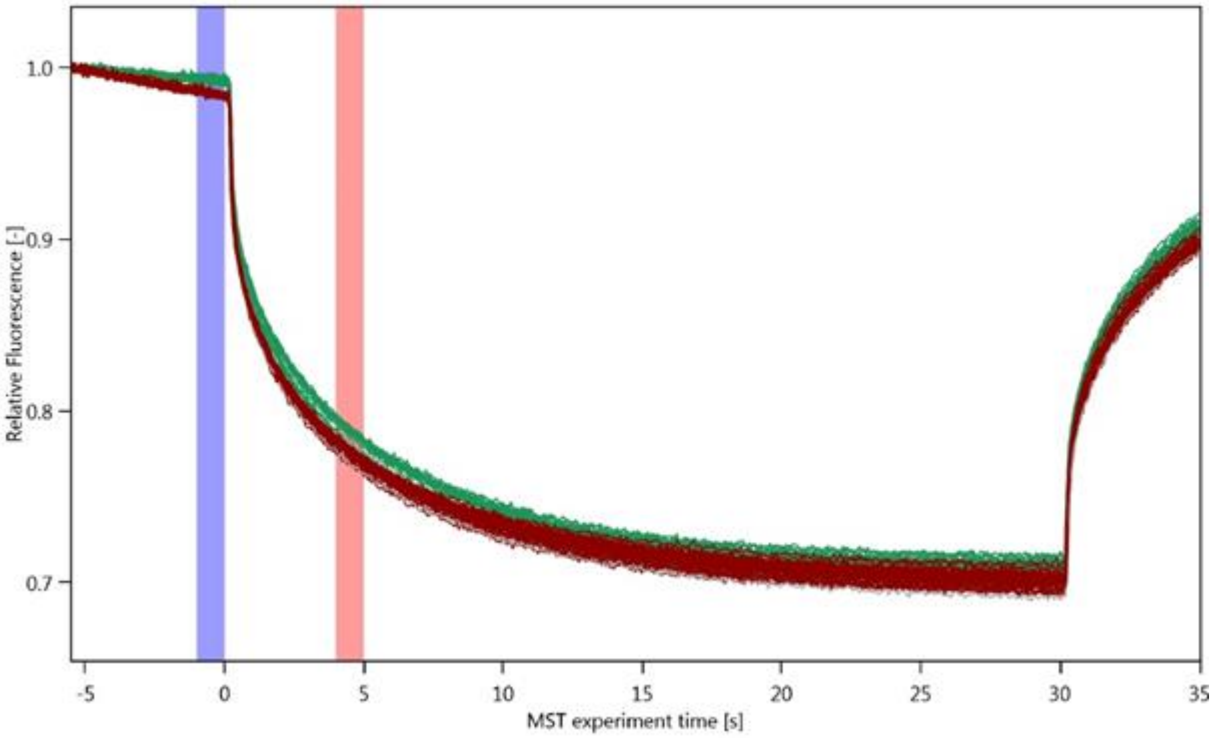

Supplementary Figure 6A| MST traces of OMe24 interacting with miRNA24. Relative Fluorescence (RF) between the bound and unbound state was determined over a time period of 35 s with 30 s MST-on time for evaluation. The blue bar indicates the ΔRF before Temperature gradient of 2.5 K was applied, whereas the red bar shows the ΔRF during the thermophoresis. Green traces: OMe24 vs. miRNA24; red traces: OMeScr vs. miRNA24.

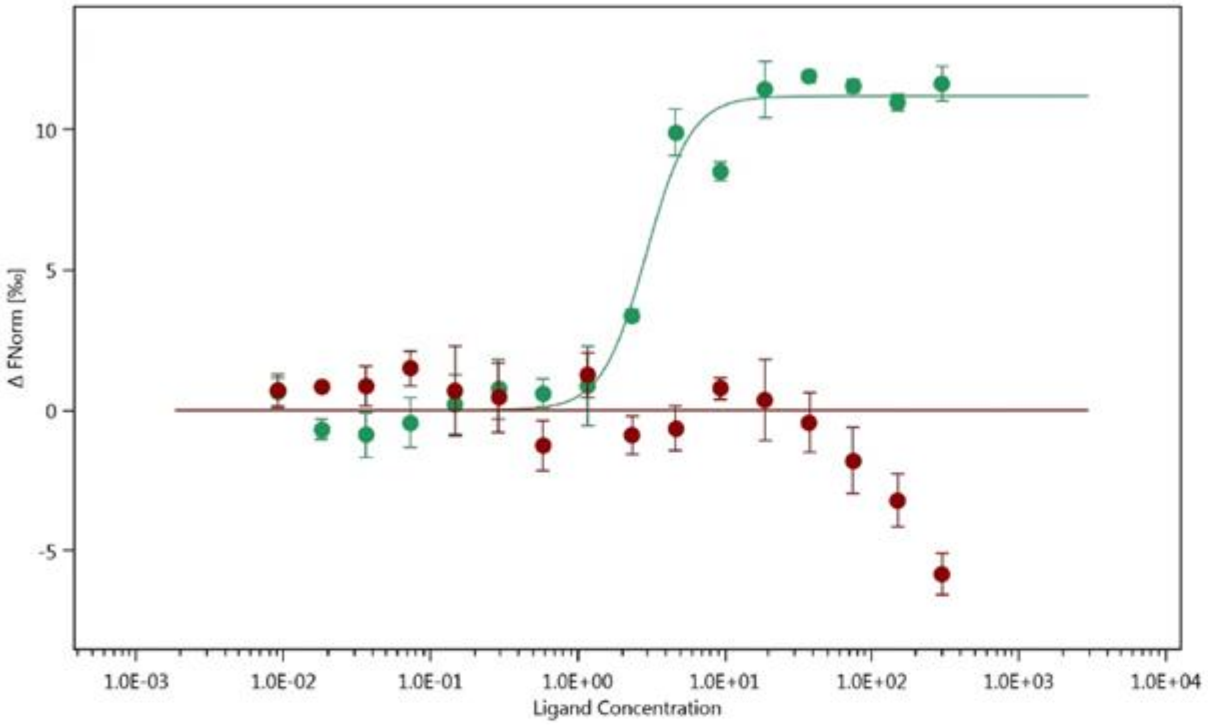

Supplementary Figure 6B| Interaction between OMe24 and miRNA24. MiRNA24 was labeled with Cy5 and used at a concentration of 3 nM while OMe24 was titrated in concentrations between 300 and 0.0092 nM. A EC_{50} of 2.944 nM +/- 0.328 nM was determined for this interaction employing standard data analysis with MO.Affinity Analysis Software. The interaction was plotted against OMeScr vs. miRNA24 as negative control. The graphs display data from 3 independent measurements. Error bars represent the standard deviation. Green dots: OMe24 vs. miRNA24; red dots: OMeScr vs. miRNA24.

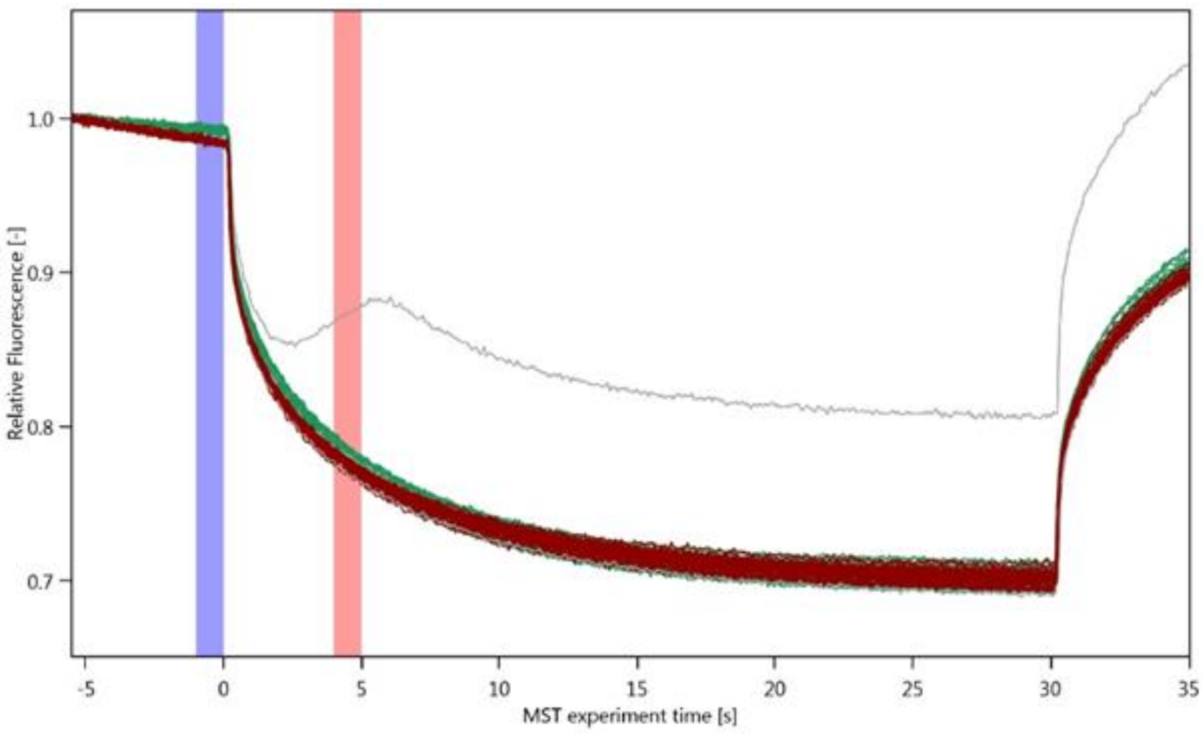

Supplementary Figure 7A| MST traces of OMe24_1 interacting with miRNA24. Relative Fluorescence (RF) between the bound and unbound state was determined over a time period of 35 s with 30 s MST-on time for evaluation. The blue bar indicates the ΔRF before Temperature gradient of 2.5 K was applied, whereas the red bar shows the ΔRF during the thermophoresis. Green traces: OMe24_1 vs. miRNA24; red traces: OMeScr vs. miRNA24.

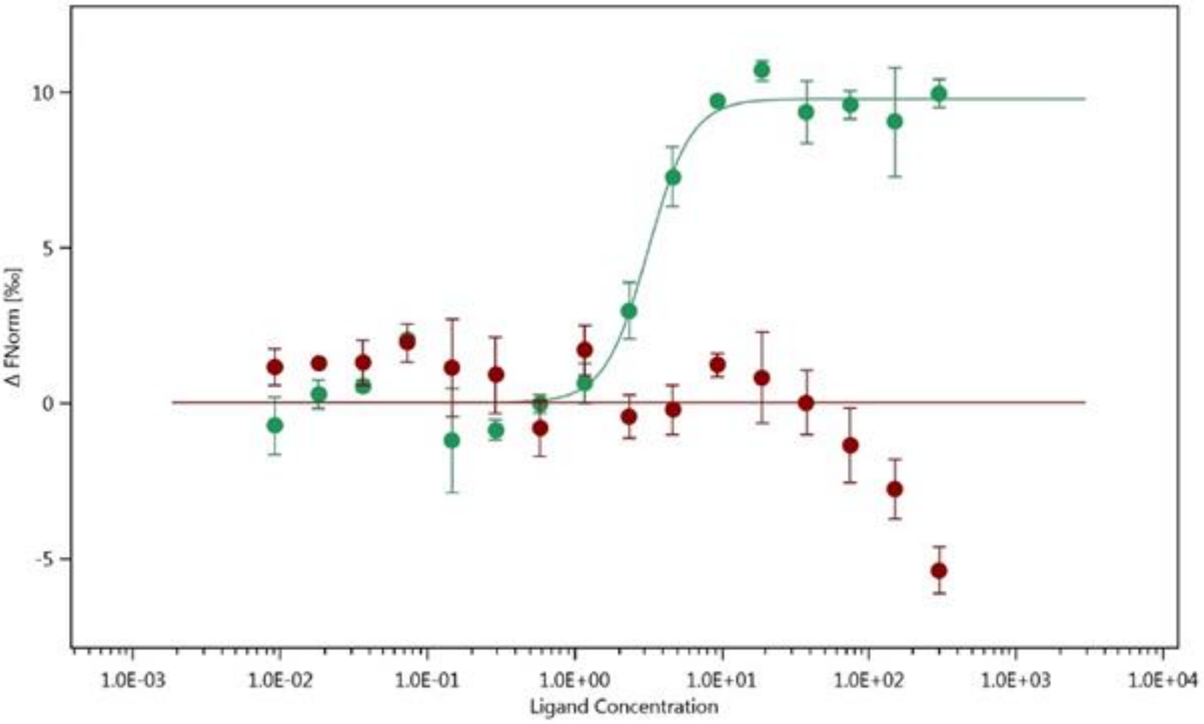

Supplementary Figure 7B| Interaction between OMe24_1 and miRNA24. MiRNA24 was labeled with Cy5 and used at a concentration of 3 nM while OMe24_1 was titrated in concentrations between 300 and 0.0092 nM. A EC_{50} of 3.153 nM +/- 0.378 nM was determined for this interaction employing standard data analysis with MO.Affinity Analysis Software. The interaction was plotted against OMeScr vs. miRNA24 as negative control. The graphs display data from 3 independent measurements. Error bars represent the standard deviation. Green dots: OMe24_1 vs. miRNA24; red dots: OMeScr vs. miRNA24.

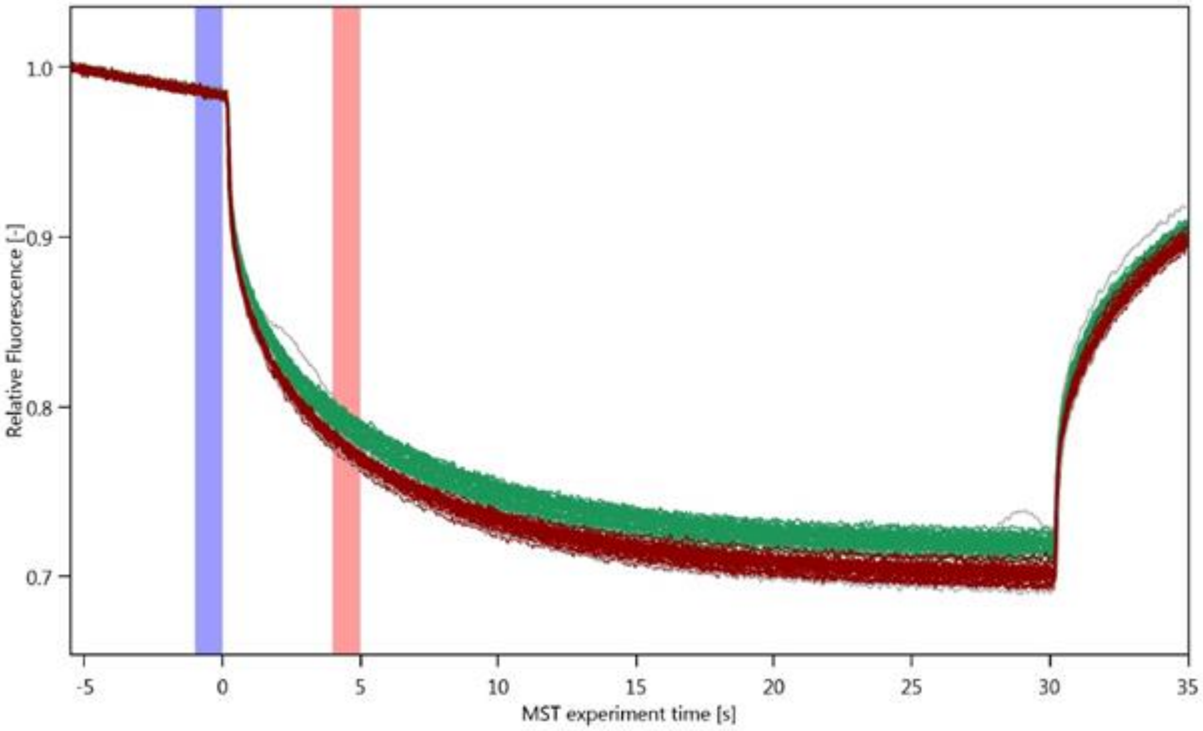

Supplementary Figure 8A| MST traces of OMe24_2 interacting with miRNA24. Relative Fluorescence (RF) between the bound and unbound state was determined over a time period of 35 s with 30 s MST-on time for evaluation. The blue bar indicates the ΔRF before Temperature gradient of 2.5 K was applied, whereas the red bar shows the ΔRF during the thermophoresis. Green traces: OMe24_2 vs. miRNA24; red traces: OMeScr vs. miRNA24.

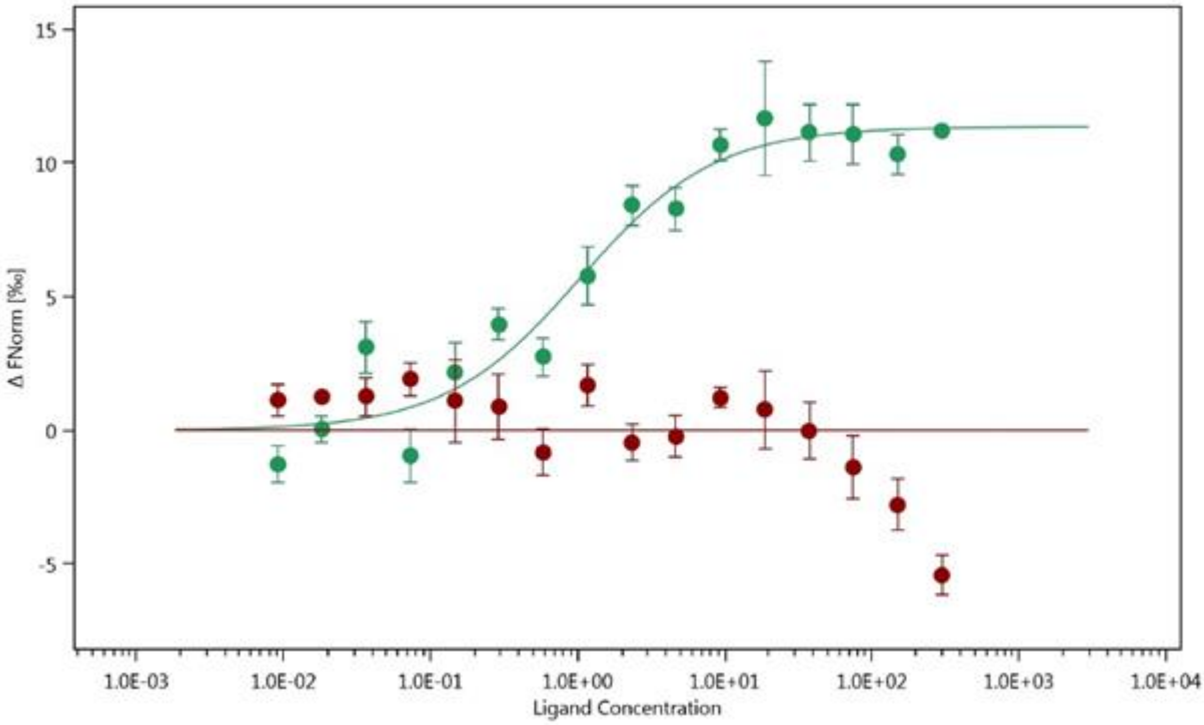

Supplementary Figure 8B| Interaction between OMe24_2 and miRNA24. MiRNA24 was labeled with Cy5 and used at a concentration of 3 nM while OMe24_2 was titrated in concentrations between 300 and 0.0092 nM. A EC_{50} of 1.039 nM +/- 0.377 nM was determined for this interaction employing standard data analysis with MO.Affinity Analysis Software. The interaction was plotted against OMeScr vs. miRNA24 as negative control. The graphs display data from 3 independent measurements. Error bars represent the standard deviation. Green dots: OMe24_2 vs. miRNA24; red dots: OMeScr vs. miRNA24.

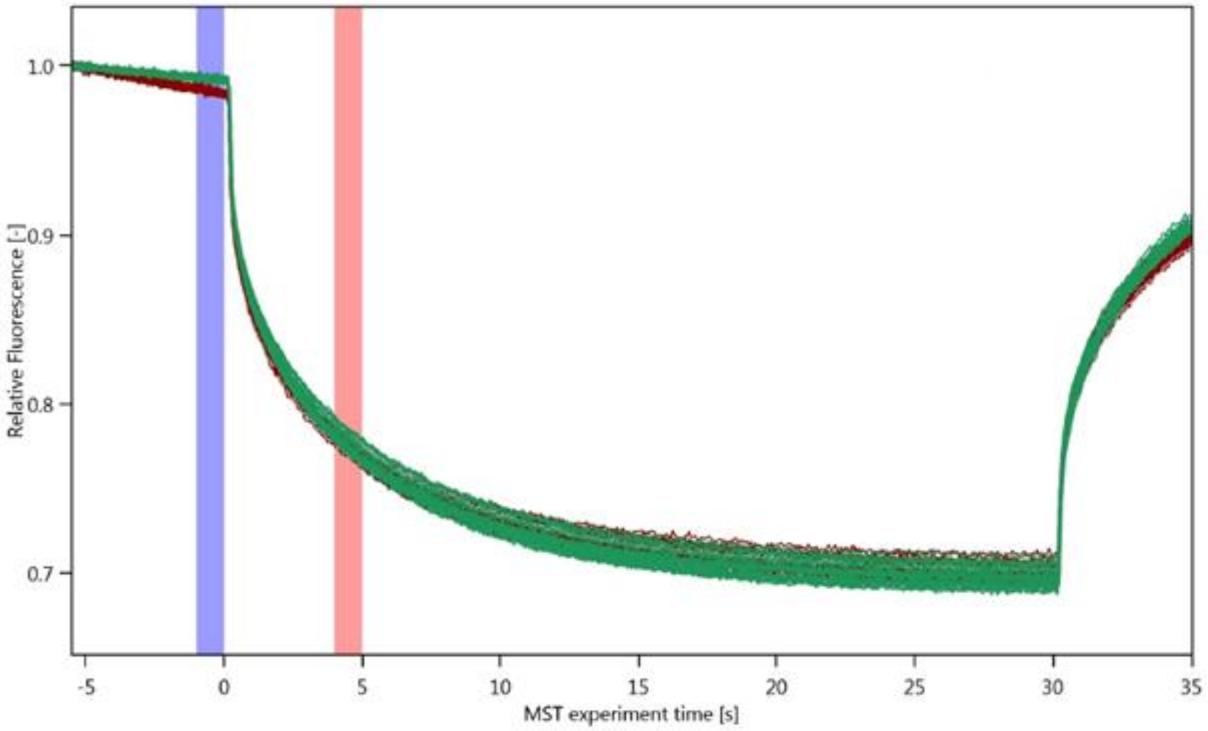

Supplementary Figure 9A| MST traces of OMe24_3 interacting with miRNA**24.** Relative Fluorescence (RF) between the bound and unbound state was determined over a time period of 35 s with 30 s MST-on time for evaluation. The blue bar indicates the ΔRF before Temperature gradient of 2.5 K was applied, whereas the red bar shows the ΔRF during the thermophoresis. Green traces: OMe24_3 vs. miRNA24; red traces: OMeScr vs. miRNA24.

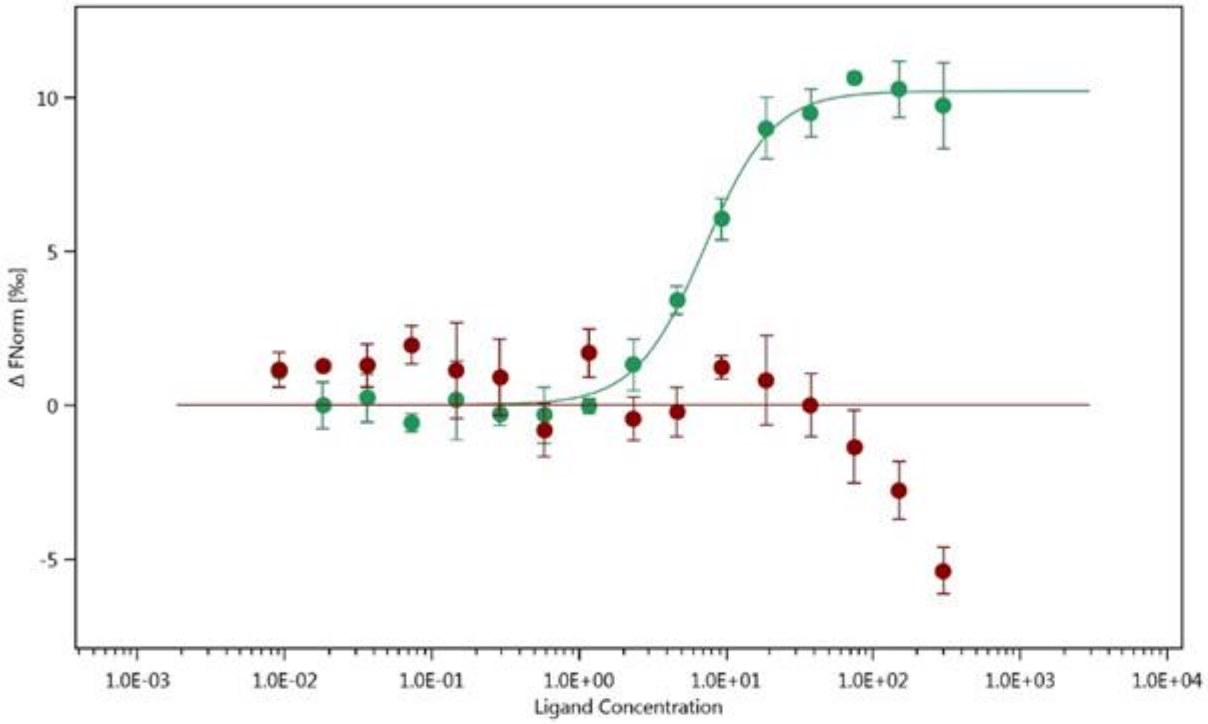

Supplementary Figure 9B| Interaction between OMe24_3 and miRNA24. MiRNA24 was labeled with Cy5 and used at a concentration of 3 nM while OMe24_3 was titrated in concentrations between 300 and 0.0092 nM. A EC_{50} of 7.155 nM +/- 0.622 nM was determined for this interaction employing standard data analysis with MO.Affinity Analysis Software. The interaction was plotted against OMeScr vs. miRNA24 as negative control. The graphs display data from 3 independent measurements. Error bars represent the standard deviation. Green dots: OMe24_3 vs. miRNA24; red dots: OMeScr vs. miRNA24.

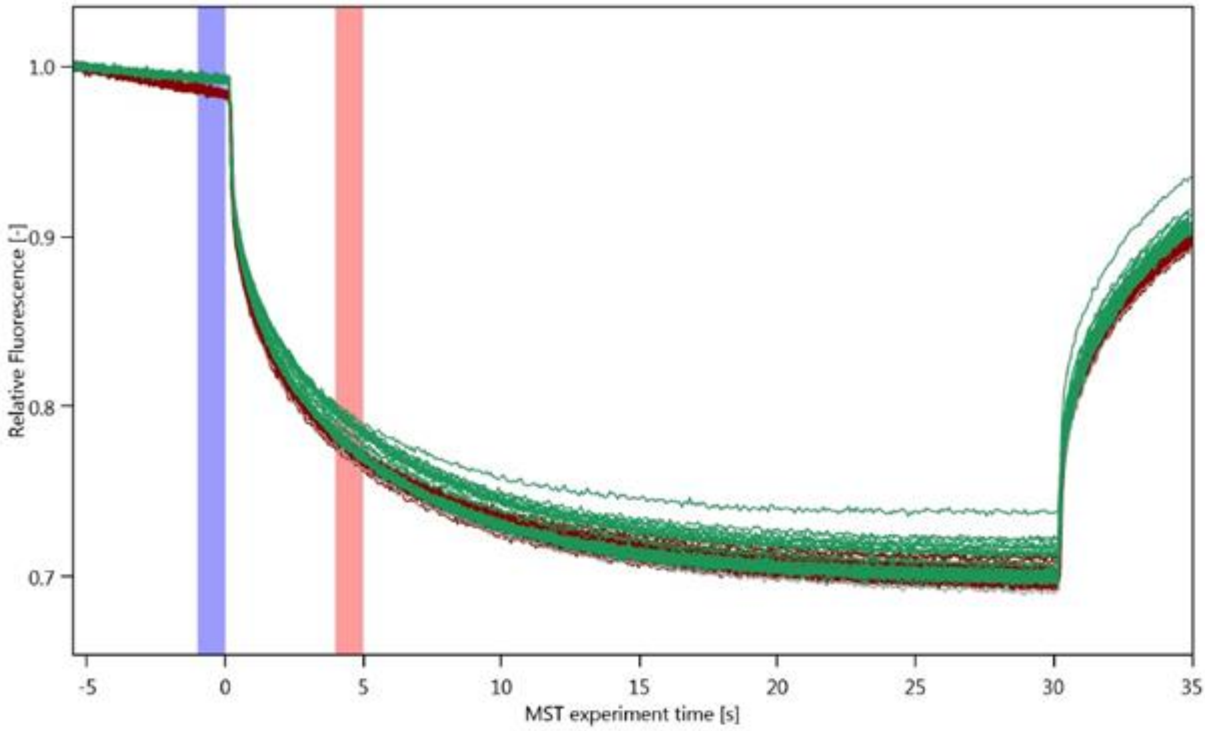

Supplementary Figure 10A| MST traces of OMe24_4 interacting with miRNA24. Relative Fluorescence (RF) between the bound and unbound state was determined over a time period of 35 s with 30 s MST-on time for evaluation. The blue bar indicates the ΔRF before Temperature gradient of 2.5 K was applied, whereas the red bar shows the ΔRF during the thermophoresis. Green traces: OMe24_4 vs. miRNA24; red traces: OMeScr vs. miRNA24.

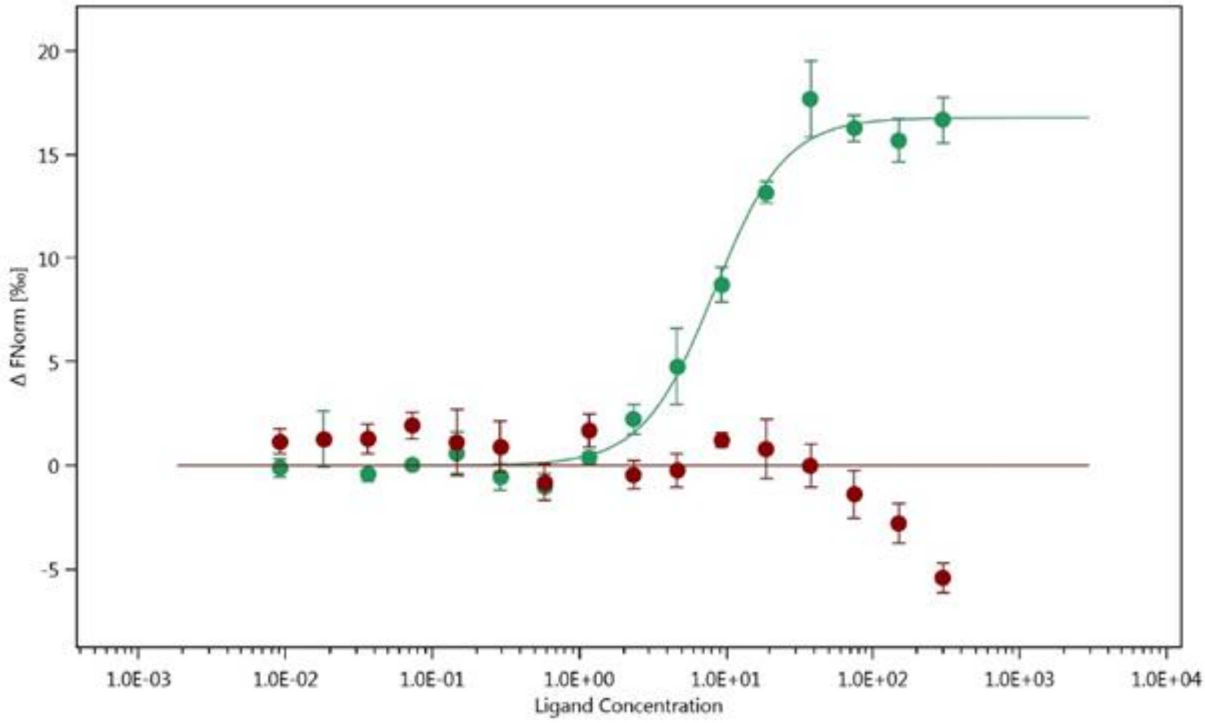

Supplementary Figure 10B| Interaction between OMe24_4 and miRNA24. MiRNA24 was labeled with Cy5 and used at a concentration of 3 nM while OMe24_4 was titrated in concentrations between 300 and 0.0092 nM. A EC_{50} of 8.318 nM +/- 0.848 nM was determined for this interaction employing standard data analysis with MO.Affinity Analysis Software. The interaction was plotted against OMeScr vs. miRNA24 as negative control. The graphs display data from 3 independent measurements. Error bars represent the standard deviation. Green dots: OMe24_4 vs. miRNA24; red dots: OMeScr vs. miRNA24.

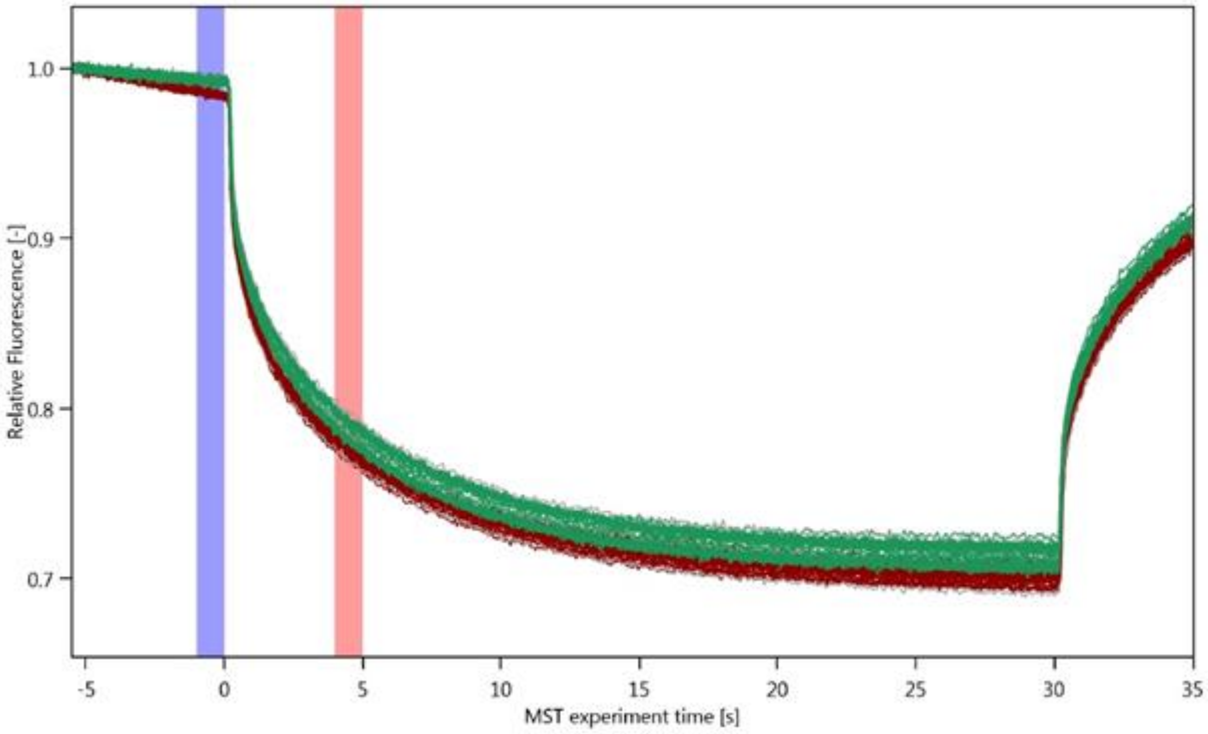

Supplementary Figure 11A| MST traces of OMe24_6 interacting with miRNA24. Relative Fluorescence (RF) between the bound and unbound state was determined over a time period of 35 s with 30 s MST-on time for evaluation. The blue bar indicates the ΔRF before Temperature gradient of 2.5 K was applied, whereas the red bar shows the ΔRF during the thermophoresis. Green traces: OMe24_6 vs. miRNA24; red traces: OMeScr vs. miRNA24.

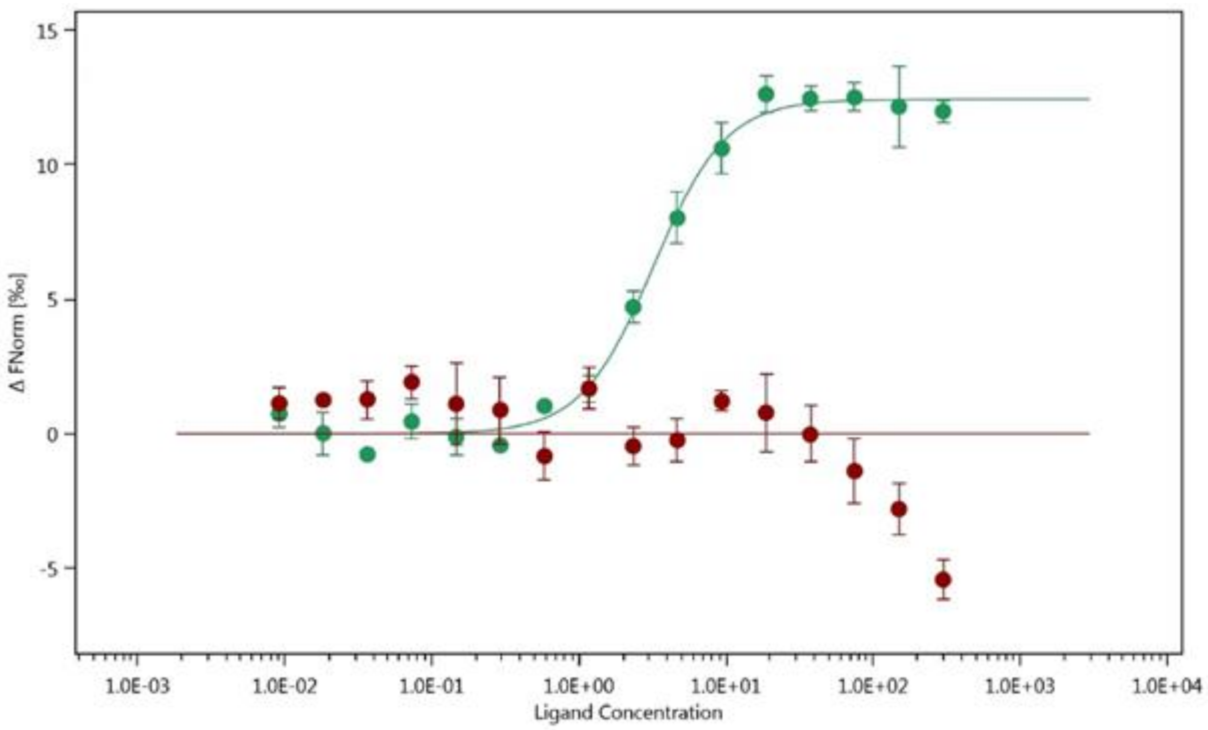

Supplementary Figure 11B| Interaction between OMe24_6 and miRNA24. MiRNA24 was labeled with Cy5 and used at a concentration of 3 nM while OMe24_6 was titrated in concentrations between 300 and 0.0092 nM. A EC_{50} of 3.216 nM +/- 0.235 nM was determined for this interaction employing standard data analysis with MO.Affinity Analysis Software. The interaction was plotted against OMeScr vs. miRNA24 as negative control. The graphs display data from 3 independent measurements. Error bars represent the standard deviation. Green dots: OMe24_6 vs. miRNA24; red dots: OMeScr vs. miRNA24.

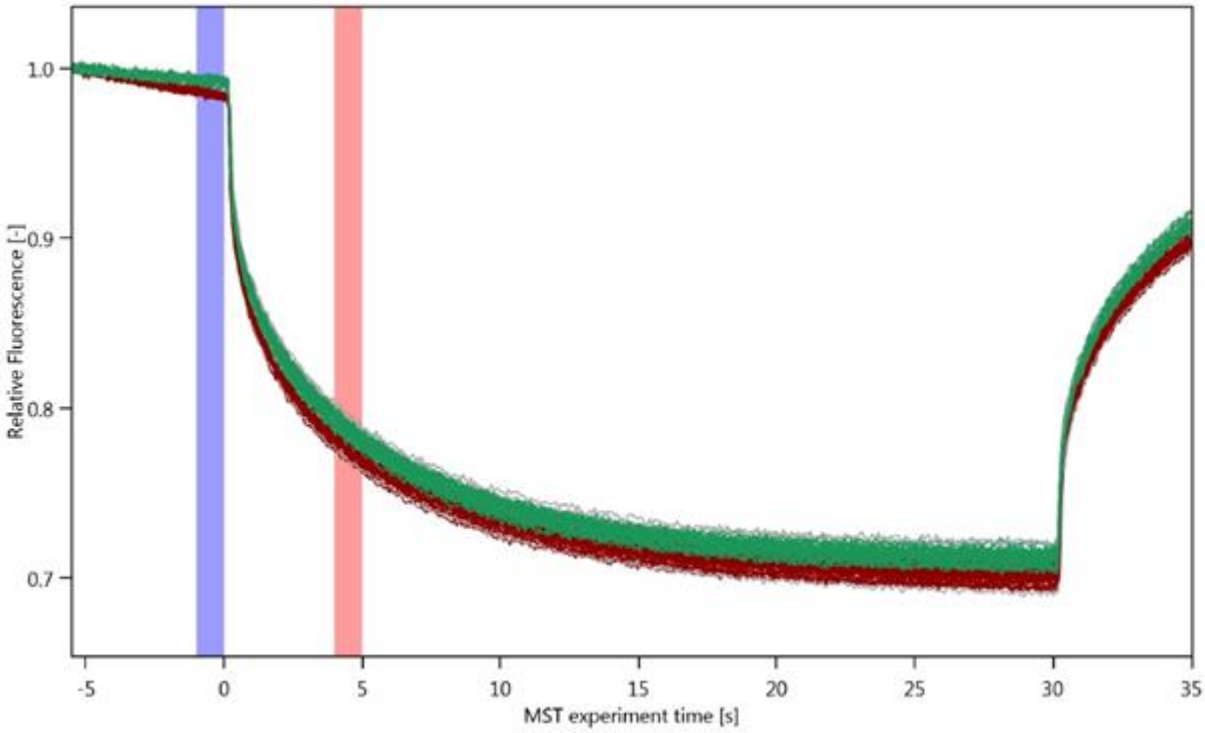

Supplementary Figure 12A| MST traces of OMe24_7 incubated with miRNA24. Relative Fluorescence (RF) between the bound and unbound state was determined over a time period of 35 s with 30 s MST-on time for evaluation. The blue bar indicates the ΔRF before Temperature gradient of 2.5 K was applied, whereas the red bar shows the ΔRF during the thermophoresis. Green traces: OMe24_7 vs. miRNA24; red traces: OMeScr vs. miRNA24.

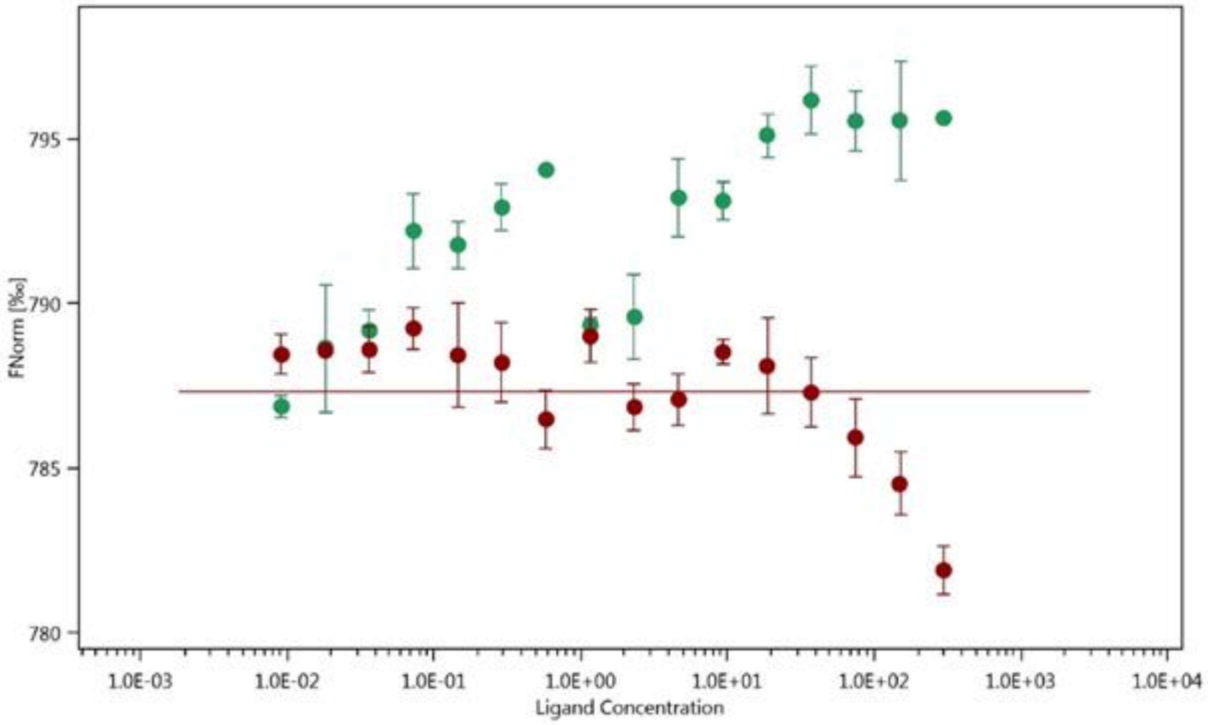

Supplementary Figure 12B| No interaction between OMe24_7 and miRNA24. MiRNA24 was labeled with Cy5 and used at a concentration of 3 nM while OMe24_7 was titrated in concentrations between 300 and 0.0092 nM. No EC_{50} could be determined. The interaction was plotted against OMeScr vs. miRNA24 as negative control. The graphs display data from three independent measurements. Error bars represent the standard deviation. Green dots: OMe24_7 vs. miRNA24; red dots: OMeScr vs. miRNA24.

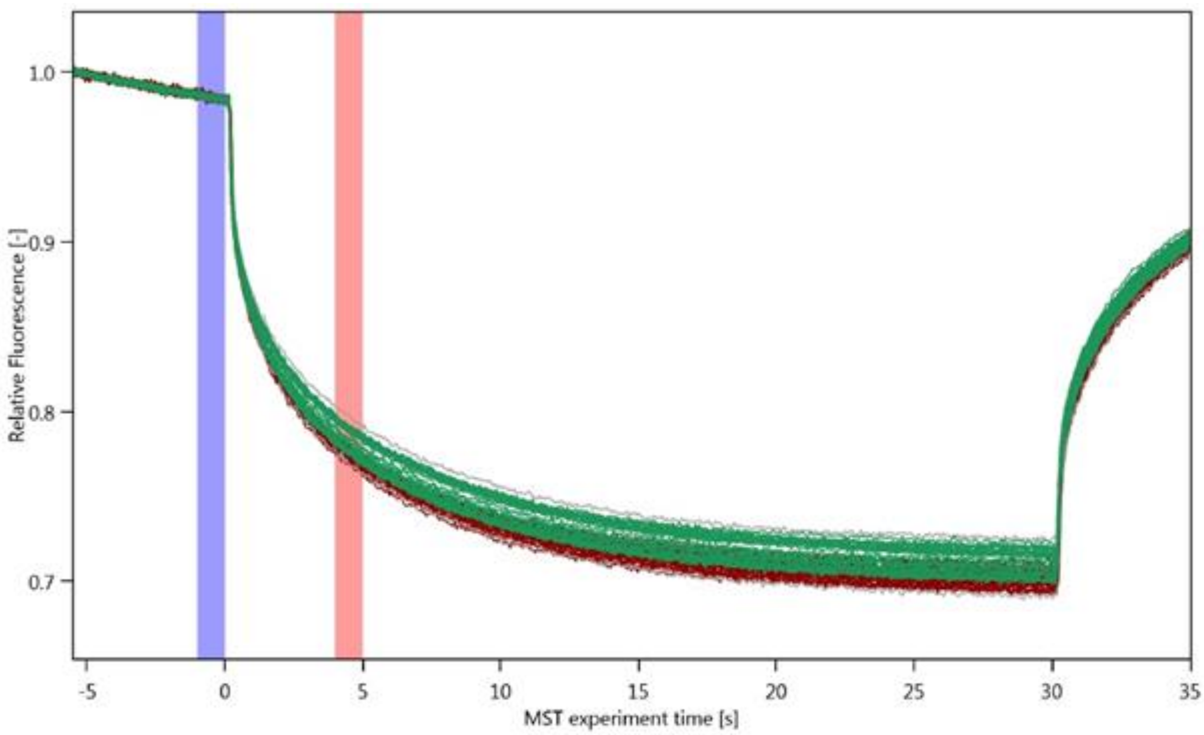

Supplementary Figure 13A| MST traces of OMe24_8 interacting with miRNA24. Relative Fluorescence (RF) between the bound and unbound state was determined over a time period of 35 s with 30 s MST-on time for evaluation. The blue bar indicates the ΔRF before Temperature gradient of 2.5 K was applied, whereas the red bar shows the ΔRF during the thermophoresis. Green traces: OMe24_8 vs. miRNA24; red traces: OMeScr vs. miRNA24.

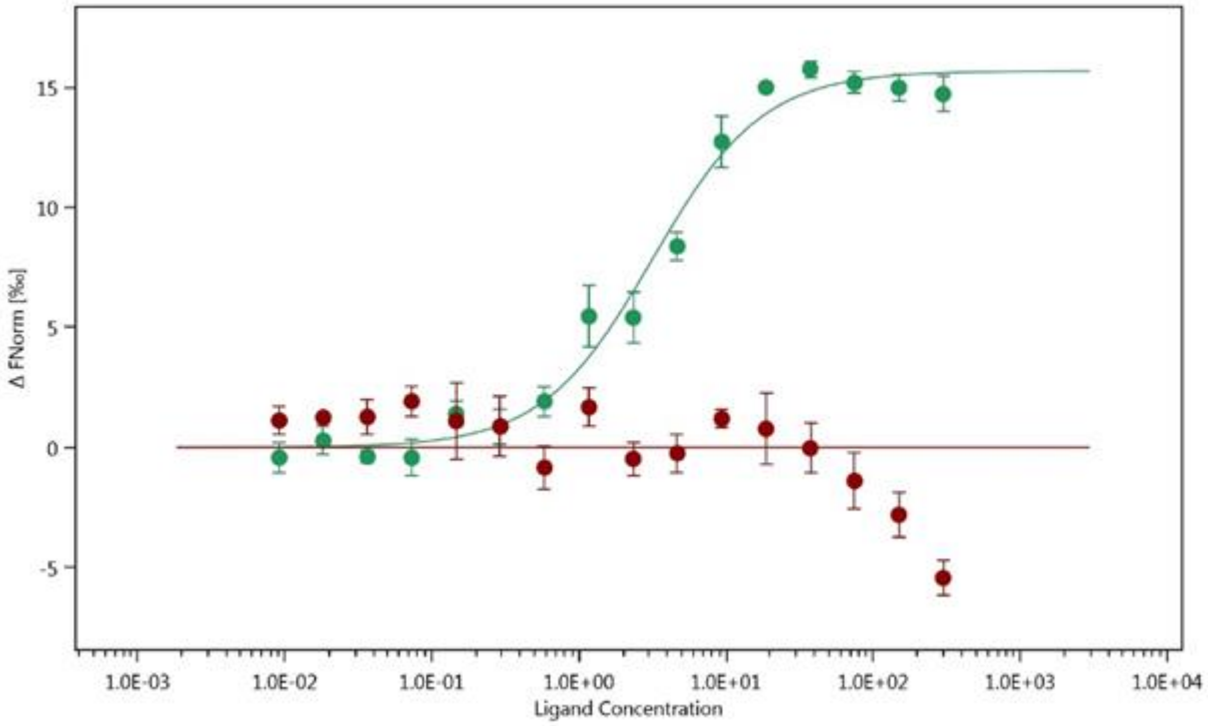

Supplementary Figure 13B| Interaction between OMe24_8 and miRNA24. MiRNA24 was labeled with Cy5 and used at a concentration of 3 nM while OMe24_8 was titrated in concentrations between 300 and 0.0092 nM. A EC_{50} of 3.162 nM +/- 0.500 nM was determined for this interaction employing standard data analysis with MO.Affinity Analysis Software. The interaction was plotted against OMeScr vs. miRNA24 as negative control. The graphs display data from 3 independent measurements. Error bars represent the standard deviation. Green dots: OMe24_8 vs. miRNA24; red dots: OMeScr vs. miRNA24.

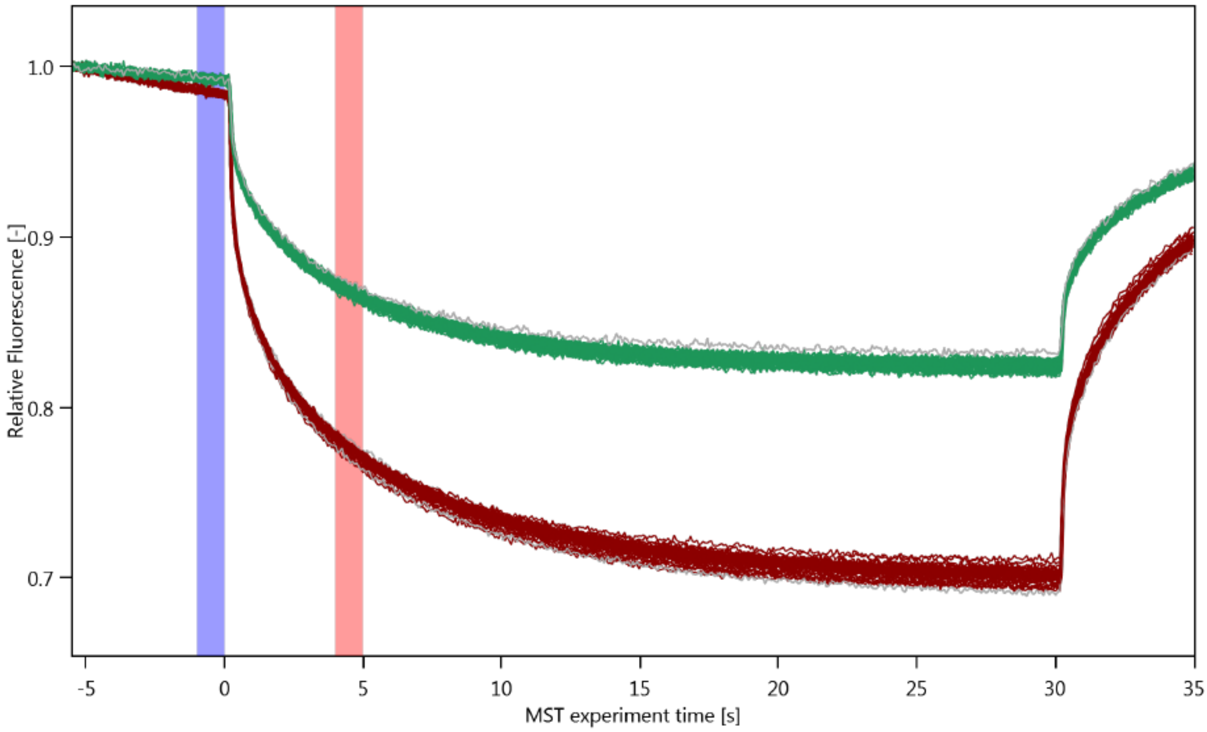

Supplementary Figure 14A| MST traces of OMe24_9 incubated with miRNA24. Relative Fluorescence (RF) between the bound and unbound state was determined over a time period of 35 s with 30 s MST-on time for evaluation. The blue bar indicates the ΔRF before Temperature gradient of 2.5 K was applied, whereas the red bar shows the ΔRF during the thermophoresis. Green traces: OMe24_9 vs. miRNA24; red traces: OMeScr vs. miRNA24.

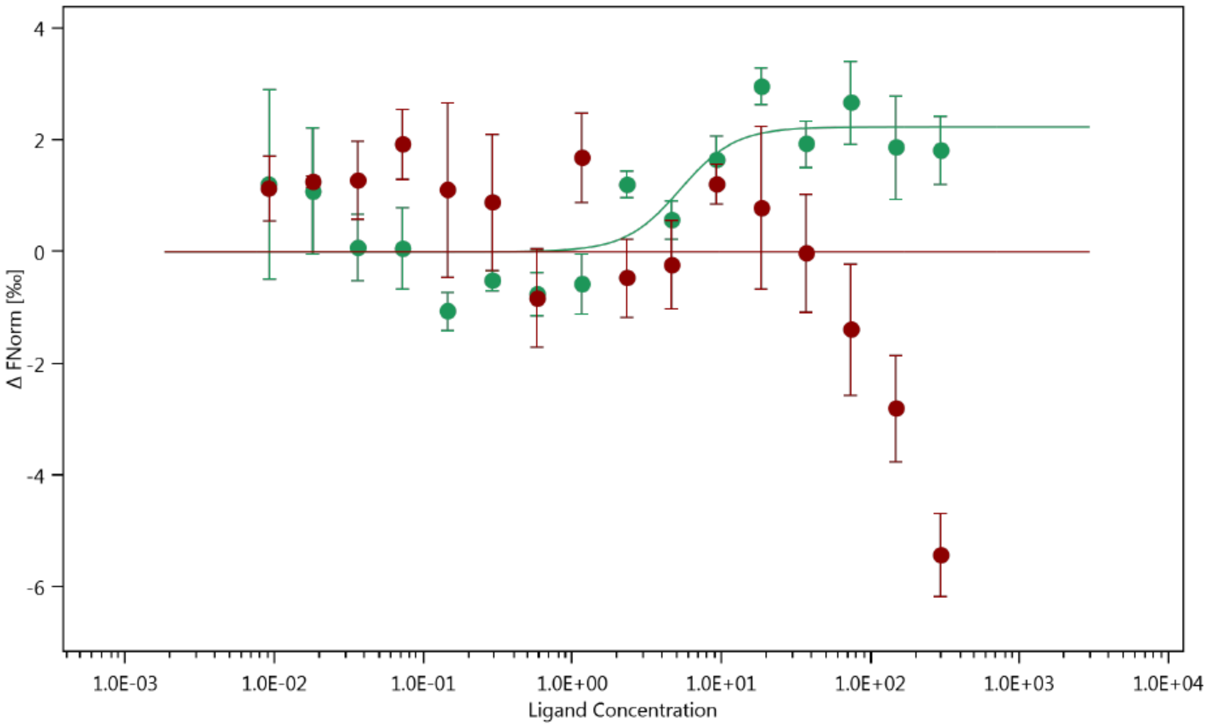

Supplementary Figure 14B| Possible interaction between OMe24_9 and miRNA24. MiRNA24 was labeled with Cy5 and used at a concentration of 3 nM while OMe24_9 was titrated in concentrations between 300 and 0.0092 nM. A EC_{50} of 5.31 nM +/- 2.84 nM was determined for this interaction employing standard data analysis with MO.Affinity Analysis Software. The interaction was plotted against OMeScr vs. miRNA24 as negative control. The graphs display data from 3 independent measurements. Error bars represent the standard deviation. Green dots: OMe24_9 vs. miRNA24; red dots: OMeScr vs. miRNA24.

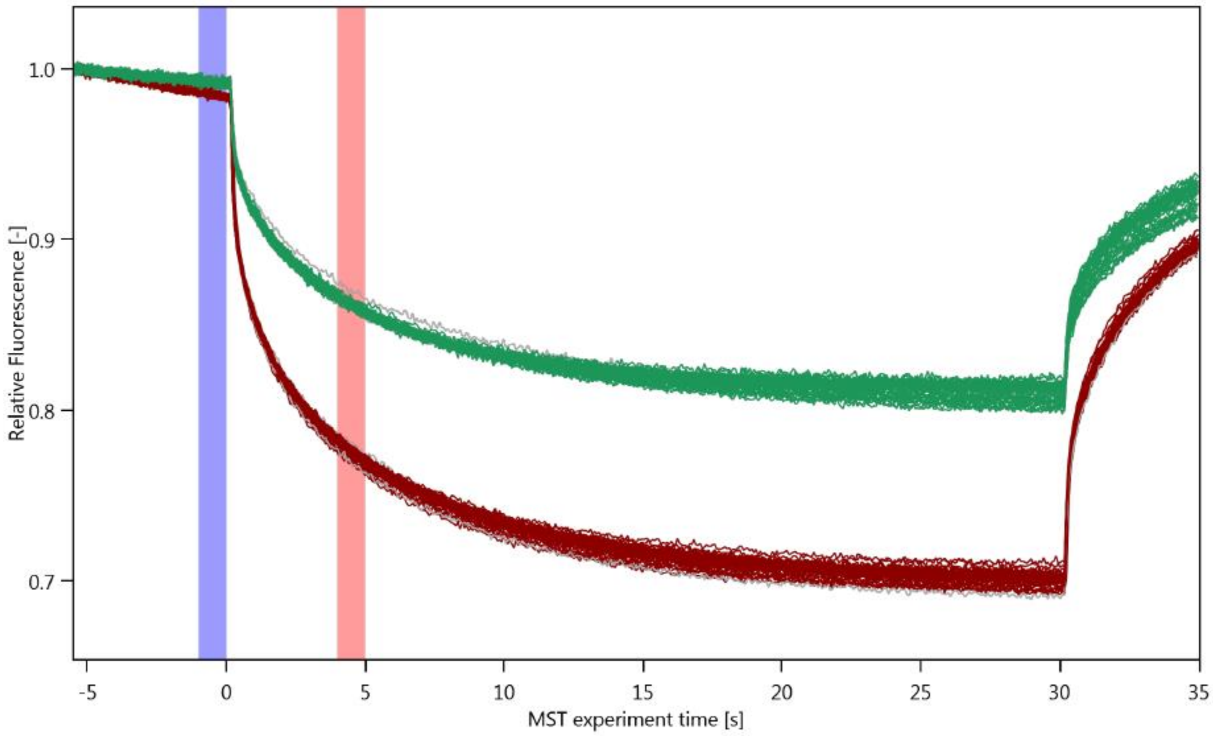

Supplementary Figure 15A| MST traces of OMe24_10 incubated with miRNA24. Relative Fluorescence (RF) between the bound and unbound state was determined over a time period of 35 s with 30 s MST-on time for evaluation. The blue bar indicates the ΔRF before Temperature gradient of 2.5 K was applied, whereas the red bar shows the ΔRF during the thermophoresis. Green traces: OMe24_10 vs. miRNA24; red traces: OMeScr vs. miRNA24.

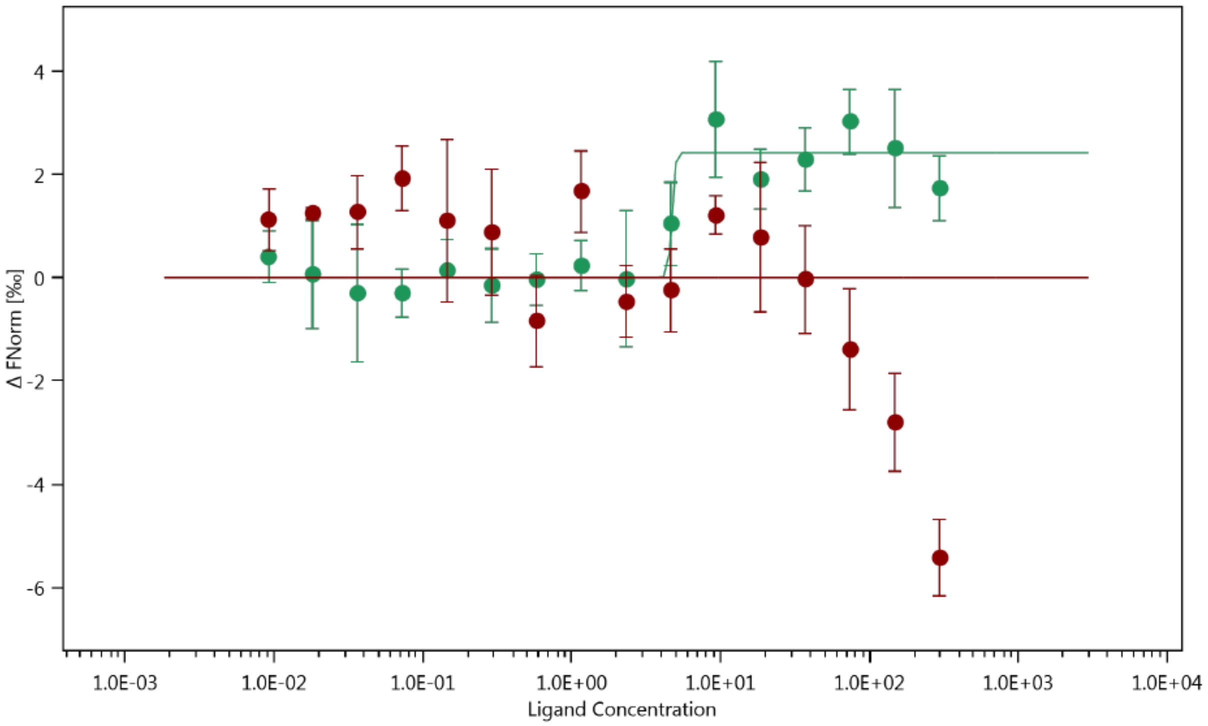

Supplementary Figure 15B| No interaction between OMe24_10 and miRNA24. MiRNA24 has been labeled with Cy5 and used at a concentration of 3 nM while OMe24_10 was titrated in concentrations between 300 and 0.0092 nM. A EC_{50} of 4.72 nM +/- $1.82*10^{8}$ nM was determined for this interaction employing standard data analysis with MO.Affinity Analysis Software. The interaction was plotted against OMeScr vs. miRNA24 as negative control. The graphs display data from 3 independent measurements. Error bars represent the standard deviation. Green dots: OMe24_10 vs. miRNA24; red dots: OMeScr vs. miRNA24.

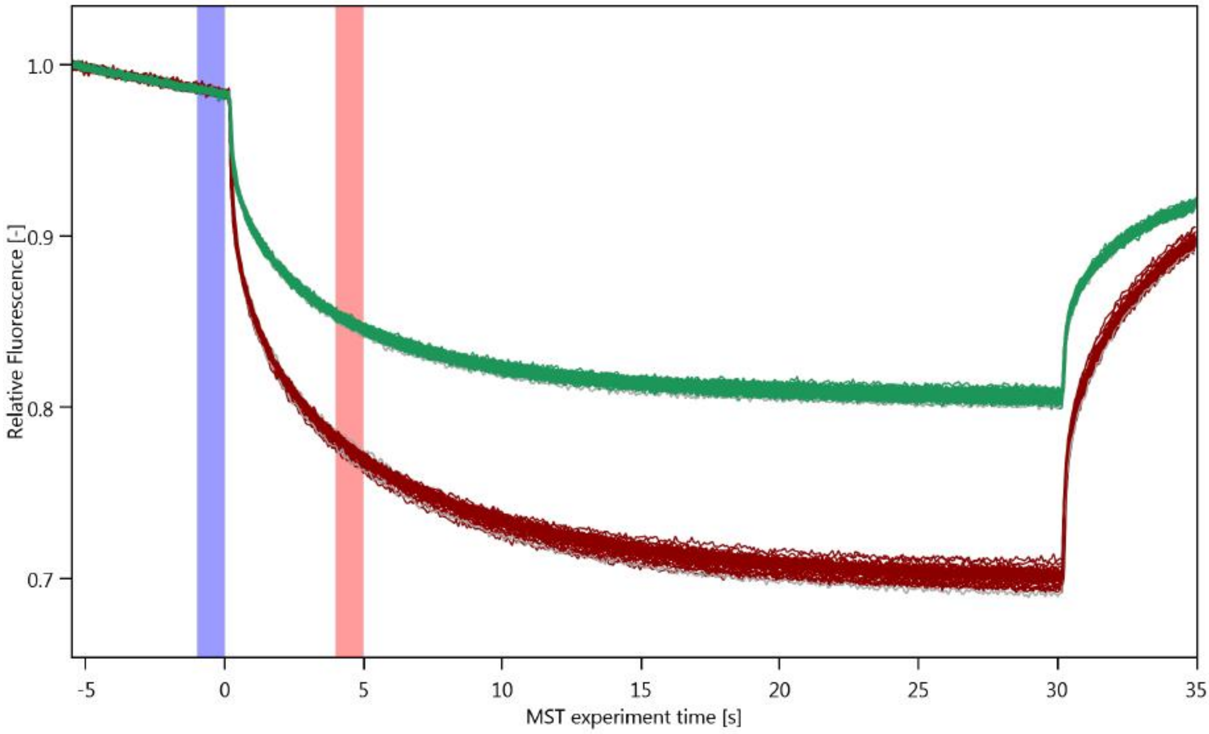

Supplementary Figure 16A| MST traces of OMe24_11 incubated with miRNA24. Relative Fluorescence (RF) between the bound and unbound state was determined over a time period of 35 s with 30 s MST-on time for evaluation. The blue bar indicates the ΔRF before Temperature gradient of 2.5 K was applied, whereas the red bar shows the ΔRF during the thermophoresis. Green traces: OMe24_11 vs. miRNA24; red traces: OMeScr vs. miRNA24.

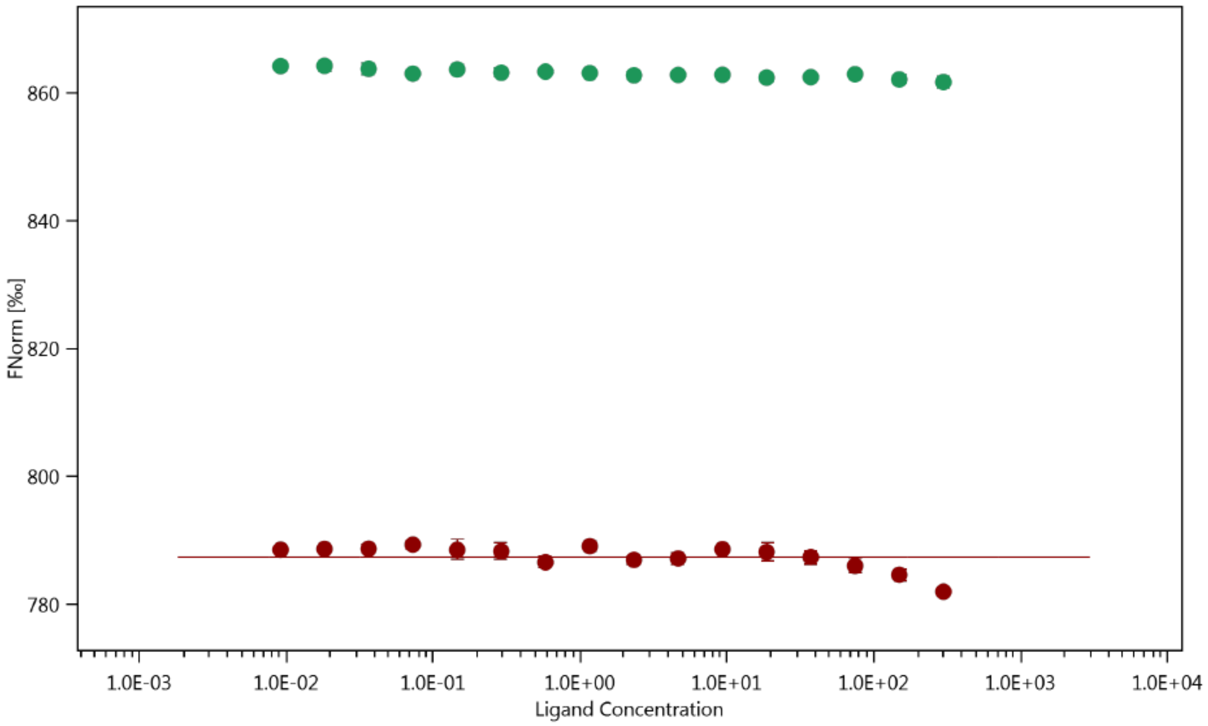

Supplementary Figure 16B| No interaction between OMe24_11 and miRNA24. MiRNA24 was labeled with Cy5 and used at a concentration of 3 nM while OMe24_11 was titrated in concentrations between 300 and 0.0092 nM. No EC_{50} could be determined. The interaction was plotted against OMeScr vs. miRNA24 as negative control. The graphs display data from 3 independent measurements. Green dots: OMe24_11 vs. miRNA24; red dots: OMeScr vs. miRNA24.
